# Crystal tensor properties of magnetic materials with and without spin–orbit coupling. Application of spin point groups as approximate symmetries

**DOI:** 10.1107/S2053273325004127

**Published:** 2025-06-10

**Authors:** Jesus Etxebarria, J. Manuel Perez-Mato, Emre S. Tasci, Luis Elcoro

**Affiliations:** a Department of Physics, Faculty of Science and Technology, University of the Basque Country/Euskal Herriko Unibertsitatea (UPV/EHU), Bilbao, Spain; b Faculty of Science and Technology, University of the Basque Country/Euskal Herriko Unibertsitatea (UPV/EHU), Bilbao, Spain; c Department of Physics Engineering Hacettepe University 06800 Ankara Türkiye; Brigham Young University, USA

**Keywords:** spin point groups, magnetic point groups, symmetry-adapted tensor forms, spin–orbit coupling

## Abstract

Spin space groups, formed by operations where the rotation of the spins is independent of the accompanying operation acting on the crystal structure, are appropriate groups to describe the symmetry of magnetic structures with null spin–orbit coupling. Their corresponding spin point groups are the symmetry groups to be considered for deriving the symmetry constraints on the form of the crystal tensor properties of such idealized structures. These groups can also be taken as approximate symmetries (with some restrictions) of real magnetic structures, where spin–orbit coupling and magnetic anisotropy are however present. Here we formalize the invariance transformation properties that must satisfy the most important crystal tensors under a spin point group. This is done using modified Jahn symbols, which generalize those applicable to ordinary magnetic point groups [Gallego *et al.* (2019). *Acta Cryst.* A**75**, 438–447]. The analysis includes not only equilibrium tensors, but also transport, optical and non-linear optical susceptibility tensors. The constraints imposed by spin collinearity and coplanarity within the spin group formalism on a series of representative tensors are discussed and compiled. As illustrative examples, the defined tensor invariance equations have been applied to some known magnetic structures, showing the differences in the symmetry-adapted form of some relevant tensors, when considered under the constraints of its spin point group or its magnetic point group. This comparison, with the spin point group implying additional constraints in the tensor form, can allow one to distinguish those magnetic-related properties that can be solely attributed to spin–orbit coupling from those that are expected even when spin–orbit coupling is negligible.

## Abstract 2

We have studied the constraints that the spin group symmetry imposes on the most important crystal tensors, on the basis of a generalization of the Neumann principle to spin point groups. Some examples of real materials are presented, and their tensor forms under the spin and magnetic point groups are compared.

## 1. Introduction

Although the theory of spin space groups (SpSGs) was proposed and developed more than 50 years ago (Brinkman & Elliott, 1966 ▸; Litvin & Opechowski, 1974 ▸; Litvin, 1977 ▸), it is only recently that these groups have become the object of much interest and have been intensively applied in the framework of electronic band studies of magnetic materials. As symmetry groups associated with negligible spin–orbit coupling (SOC), the SpSG of a magnetic structure is in general a supergroup of its magnetic space group (MSG), and as a consequence the SpSG may dictate symmetry constraints on the properties of the material, additional to those resulting from its MSG. In the framework of electronic bands, more symmetry constraints in general imply more band degeneracies. Thus, the application of SpSGs has been used to identify the presence of spin band splittings, which are present not only under the MSG of the structure but also under its SpSG, being therefore quite robust and especially important as SOC-free effects (Liu *et al.*, 2022 ▸). For example, the so-called altermagnets, which refer to collinear antiferromagnets with spin splitting in the SOC-free limit (Yuan *et al.*, 2020 ▸; Šmejkal *et al.*, 2022*a* ▸; Šmejkal *et al.*, 2022*b* ▸; Mazin, 2022 ▸), can be described in terms of their SpSGs. The same can be said for other forms of unconventional magnetism in materials with non-collinear magnetism (Yuan *et al.*, 2021 ▸; Hellenes *et al.*, 2024 ▸). It is in this context that three independent groups have very recently enumerated and classified the SpSGs, and considered in detail their application in the symmetry analysis of electronic bands of magnetic materials (Chen *et al.*, 2024 ▸; Jiang *et al.*, 2024 ▸; Xiao *et al.*, 2024 ▸).

In general, the comparison of the SpSG and the MSG of a magnetic structure could be used to distinguish and resolve features and properties that are only SOC effects, and therefore they are expected in general to be quite weak or even negligible. In practice, for real materials, this approach may partially fail if the observed spin arrangement includes features due to SOC effects. Notwithstanding this problem, the relevance of SpSGs in the study of tensor properties of magnetic materials, establishing a general rigorous formalism, is still a field to be explored in detail. Some recent contributions have already been made (Watanabe *et al.*, 2024 ▸; Zhu *et al.*, 2024 ▸). This work is a further step in this direction.

Gallego *et al.* (2019 ▸) carried out a comprehensive analysis of the symmetry-adapted form of all kinds of crystal tensor properties in non-magnetic and magnetic materials, considering their relevant symmetry groups, namely crystallographic point groups and crystallographic magnetic point groups, respectively. Here, following a similar approach, we analyze the symmetry-adapted forms of crystal tensors under the spin point group (SpPG) associated with the SpSG of a structure, and compare them with those expected from its actual magnetic point group (MPG).

The article is organized in the following form: after a recapitulation of the physical meaning and mathematical structure of the SpSGs and their corresponding SpPGs, their relation with ordinary MPGs is discussed in detail. We then formalize the symmetry conditions to be satisfied by crystal tensors in magnetic crystals under a given SpPG. For this purpose, the Jahn symbols (Jahn, 1949 ▸), describing the transformation properties of each tensor for the symmetry operations, are here generalized to take into account the particular features of SpPG operations. Using this generalization, we establish the corresponding generalized Jahn symbol for all kinds of tensors, including equilibrium, transport and optical properties. This formalism is then applied to a series of examples of experimental magnetic structures, for which the symmetry-adapted form of various tensors under the SpPG of the structure is determined, and compared with the less stringent constraints under its MPG, where possible SOC effects are necessarily taken into account. Very different types of SpPG–MPG relations can be realized in a magnetic structure, and the examples presented here try to cover the most representative ones. Finally, in Appendix *A*[App appa] we have included a glossary of some important groups used in the paper and their notation.

## 2. Spin space groups and spin point groups

### 2.1. Spin space groups as the symmetry groups of SOC-free magnetic structures

A well defined symmetry group of a physical system must be constituted by operations which, apart from keeping the system indistinguishable, constitute a subgroup of the group of transformations that keep the energy of the system invariant. This ensures that the constraints on the system implied by these operations are *stable*, in the sense that they are maintained if, for instance, in the case of a thermodynamic system, temperature or pressure are varied (excluding a symmetry-breaking phase transition taking place); or in the case of a system ground state, the symmetry constraints are maintained if the Hamiltonian parameters are continuously varied. As a consequence, a symmetry group defined under this condition can be assigned to a whole thermodynamic phase, or to the ground state for some continuous range of the Hamiltonian parameters. This is why in non-magnetic commensurate crystal structures the operations of the space groups, which describe their symmetry, are formed by combinations of rotations, translations and space inversion, which all keep the energy invariant. Hereafter, we shall call this type of operations *space operations*, and they will be generally represented by the symbol 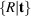, where *R* represents a proper or improper rotation of the system, including the limiting cases of *R* being the identity 1, or the space inversion 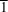, while 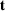 represents a space translation of the system.

In the case of incommensurate modulated crystal structures, global phase shift(s) of the incommensurate modulation(s) also keep the energy invariant and, therefore, the so-called *superspace groups* describing the symmetry of these systems are constructed by adding these extra energy-invariant transformations, when the combined symmetry operations that keep the system indistinguishable are defined (Janssen *et al.*, 2004 ▸). For instance, a generic operation of a (3 + 1)D superspace group with a single independent incommensurate wavevector can be expressed as 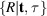, indicating that the space operation 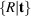 is followed by a global shift τ of the incommensurate modulation in the structure (Perez-Mato *et al.*, 1984 ▸).

In the same way, in the case of commensurate magnetic structures, MSGs are constructed by adding the time-reversal operation, which reverses both spins and momenta, when defining the operations of the group (Litvin, 2016 ▸; Campbell *et al.*, 2024 ▸). The time-reversal operation indeed keeps the energy invariant, and is in fact a trivial symmetry operation always present (and therefore not explicitly considered) in all non-magnetic or magnetically disordered structures, while in magnetic structures it may only be present if combined with some space operation different from the trivial identity. Thus, a generic operation of an MSG can be expressed as 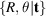, with θ being −1 if time reversal is included, and +1 otherwise. It is important in the context of the present work to stress that the space operation 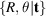 of an MSG necessarily operates on the system as a whole, *i.e.* it also includes a transformation of its atomic spins or its spin density, as the spin orientation and the crystal structure are in general energy-coupled through the SOC. Thus, an energy-invariant operation 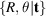 transforms not only the crystal structure, given for instance by a scalar density 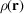, with the space operation 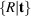: 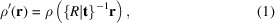
but it also transforms the magnetic moment density 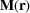 of the system into a new one 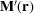 that satisfies
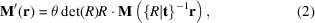
where 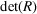 is the determinant of the matrix *R*. Thus, in equation (2), both the axial-vector character of the magnetic moment and the inclusion or not of time reversal in the operation are taken into account. If after applying the operation the transformed functions coincide with the original ones, so that 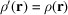 and 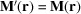, then the operation 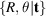 belongs to the MSG of the structure.

MSGs are therefore the appropriate groups that can describe the symmetry of a commensurate magnetic structure, *i.e.* the set of symmetry constraints that are expected to be satisfied by the structure within the whole range of a thermodynamic phase, or in the case of a ground state, to be satisfied within a continuous range of the Hamiltonian parameters. However, if the SOC in the structure can be considered negligible, then any arbitrary global rotation 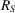 of the spin arrangement, with full independence of the crystal orientation, is also energy-invariant. Here, however, we must explicitly separate the usually small orbital contribution 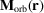 to the magnetization density 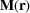 from the contribution of the actual spins 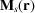, because these additional energy-free spin rotations to be included refer only to 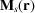, while the orbital contribution 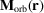 remains locked to the space operations. Hence, we can express these additional energy-invariant transformations of 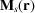 as
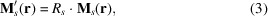
where 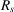 is any 3D proper rotation. This extension in SOC-free structures of the set of energy-invariant transformations implies that their symmetry can be described by the SpSGs (Brinkman & Elliott, 1966 ▸; Litvin & Opechowski, 1974 ▸), where operations of the type considered in MSGs can also be combined with spin rotation operations of the type indicated in equation (3). Thus, a generic operation of an SpSG could be expressed as 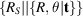, indicating the combination of an MSG-type operation 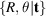 with an additional proper rotation 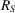 of the spins. As in SOC-free structures spins are uncoupled from the crystal structure, 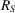 in the operation above can be defined in such a way that it includes the necessary rotation to be applied to the spins, while the space operation 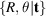, in contrast with its interpretation in an MSG, does not act on the spins, but applies only to the magnetic moments of orbital origin.

Hence, if 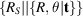 is an operation of the SpSG of a magnetic structure, it implies that the following equations are fulfilled: 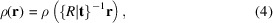

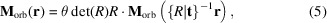

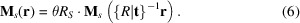
Thus, the rotation applied to the spins is fully unlocked from the space operation and can be an improper one, 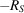, or a proper one, 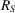, depending on whether the operation includes time reversal or not. In contrast, the atomic magnetic moments of orbital origin are locked to the crystal and are transformed in the usual form of an MSG operation.

For convenience, following the usual convention, we simplify the notation of SpSG operations in the form 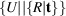, where *U* represents the proper or improper rotation 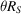 indicated in equation (6), and, therefore, if *U* is an improper rotation, the whole operation includes time reversal, and this inclusion not only applies to the spins but also to the orbital degrees of freedom in equation (5) and any other time-related variables in the system, like momenta. Hence, while the symbol 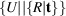 denotes the space operation part as 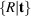, it is important to take into account that this space operation may include time reversal, depending on the value of the determinant of *U*, although it is not explicitly indicated.

In the case of an experimental magnetic structure, equation (5) for the orbital magnetic moments is difficult to assess, as orbital and spin contributions generally remain unresolved. Given the expected smallness or null value of the orbital contribution, equation (6) is usually assumed to be applicable to the determined atomic magnetic moments (Chen *et al.*, 2024 ▸; Jiang *et al.*, 2024 ▸; Xiao *et al.*, 2024 ▸). However, it should be noted that this assumption may fail, and equations (5) and (6) imply that, under the constraints of an SpSG (and therefore assuming negligible SOC), orbital atomic magnetic moments and spin moments may be forced to have different directions. This can only happen in the case of non-coplanar magnetic structures because, as explained below, the SpSGs of collinear and coplanar structures forbid, through equation (5), any magnetic ordering of orbital type (Watanabe *et al.*, 2024 ▸).

### 2.2. Subgroups of SpSGs. The non-trivial SpSG and the spin-only subgroup

Several important subgroups can be distinguished in an SpSG. The *spin-only* subgroup is formed by the operations of type 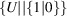, *i.e.* operations that do not involve any space operation, except the identity, or time reversal in the case that 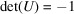. Following the notation of Chen *et al.* (2024 ▸) ▸, if we call G_SO_ the spin-only subgroup, the full SpSG, say G_SS_, can be described as the direct product of a so-called non-trivial SpSG, G_NT_, and the *spin-only* subgroup G_SO_ (Litvin & Opechowski, 1974 ▸): 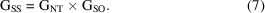
Note that, by definition, each space operation 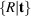 in G_NT_ is paired with one, and only one, spin operation *U*.

Only the SpSGs of collinear and coplanar structures have *spin-only* subgroups G_SO_ different from the trivial identity. Collinear structures have all the same G_SO_, formed by the continuous point group of all rotations around the direction of the spins and all mirror planes containing this direction. Similarly to Chen *et al.* (2024 ▸), we will designate this spin-only subgroup, common to all collinear structures, as 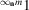. Although formally in an SpSG the collinearity direction is arbitrary with respect to the crystal lattice, for reasons explained below, we also indicate explicitly a specific orientation with respect to the lattice of the operations by means of a subscript 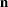.

The spin-only subgroup G_SO_ of all coplanar structures is formed by the identity and a mirror plane with the orientation of the spin planes, *i.e.*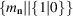, with 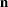 indicating the perpendicular direction to the spin planes. In an analogous manner to the collinear G_SO_, we denote the group as 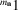, where 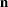 indicates a specific direction with respect to the lattice. Equation (7) implies that collinear and coplanar structures have very specific SpSGs, distinguishable by their spin-only subgroup, either 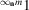 or 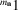. We shall call them *collinear* and *coplanar* SpSGs, respectively. It is important to stress that this formally implies that collinearity and co­planarity are always *symmetry-protected* in a SOC-free structure. We shall call all other SpSGs, which have as G_SO_ only the identity, *non-coplanar* SpSGs, since they can only be associated with magnetic structures that are neither collinear nor coplanar.

In collinear and coplanar SpSGs, their non-trivial subgroup G_NT_ defined by equation (7) is not unique. Keeping the group structure, the *U* of some of the operations 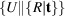 of G_NT_ can be substituted by its product with some spin operation of the corresponding spin-only subgroup 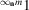 or 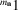. In the case of collinear structures, the non-trivial G_NT_ is usually chosen such that the *U* operations are either the identity or the inversion. This can always be done because all possible *U* operations compatible with collinearity (*i.e.* arbitrary proper or improper rotations about the spin direction 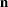, twofold axes perpendicular to 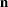 or planes containing 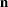) can be written as the product of a *U* operation of G_SO_ and the identity or the inversion. Thus, the G_NT_ of a collinear SpSG is isomorphic to a Shubnikov group, where each space operation is completed with a spin operation 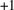 or 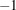, similar to what is done with ordinary MSGs. However, since in the SpSG the space operations do not act on the spins, this Shubnikov-like non-trivial SpSG is generally different from the MSG of the structure. While the non-trivial G_NT_ of a collinear SpSG, defined by a Shubnikov-like group, is independent of the spin-lattice orientation, the MSG, which is also a subgroup of the SpSG and is also described by a Shubnikov group, generally depends on the direction of the spins with respect to the crystal structure. Several examples of this situation will be discussed below.

In the case of coplanar SpSGs, by convention the non-trivial groups G_NT_ are chosen such that their spin operations *U* are all proper rotations in 3D. This choice can always be made (Litvin & Opechowski, 1974 ▸), since any improper *U* operation can be automatically transformed into a proper one by multiplying it by the mirror operation of G_SO_. In the case of non-coplanar SpSGs, the spin-only group is trivial, and the full SpSG coincides with the non-trivial subgroup.

Another important subgroup of an SpSG is formed by all operations of type 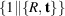, *i.e.* space operations that are not accompanied by any spin rotation, nor by time reversal. By definition, this is a subgroup of the non-trivial subgroup of the SpSG. The set of space operations 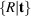 of this subgroup is an ordinary space group, say L_0_. If we call G_0_ the ordinary space group formed by all space operations 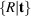 present in G_NT_, this space group G_0_ can then be decomposed in cosets with respect to L_0_: 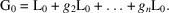
As L_0_ is a normal subgroup of G_0_ (Litvin & Opechowski, 1974 ▸), the cosets in the above equation form a factor group G_0_/L_0_ with coset representatives 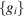. All space operations in a coset 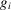
L_0_ have associated the same spin point-group operation, say 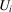. Hence, the point group formed by all spin operations 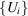 present in G_NT_ is isomorphic to the factor group G_0_/L_0_. This is a property that has been systematically applied for the enumeration of non-trivial SpSGs (Chen *et al.*, 2024 ▸; Jiang *et al.*, 2024 ▸).

The mentioned recent works that classify and enumerate SpSGs use different alternative notations, and the establishment of a unified nomenclature will still require time and effort. We will therefore not enter into notation details in this work, and when describing a specific SpSG, we will indicate its symbol in the notation proposed by Chen *et al.* (2024 ▸), complemented with a full description of a set of generators of the group, if necessary. These authors also use a four-index notation, 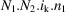, for the non-trivial part of the SpSGs, where 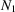 and 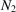 are the numerical indices in the *International tables for crystallography* (Aroyo, 2016 ▸) for the space groups L_0_ and G_0_, respectively, associated with the SpSG. The number 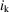 is the *klassengleich* index of L_0_ with respect to G_0_, and 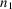 is just an ordering index. The *klassengleich* index 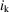 indicates the multiplication factor of a primitive unit cell describing the lattice of L_0_ with respect to that of G_0_. Therefore, if 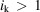, the non-trivial subgroup G_NT_ of the SpSG necessarily includes some operations of type 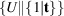, which are very important when considering the corresponding SpPG.

In an SpSG, by definition, the spin operations *U* are independent of the space operations. This has led to the convention of using an orthonormal reference system for the description of these operations, fully independent of the crystallographic axes, with its orientation only partially fixed in collinear and coplanar structures to the spin directions or the spin planes, respectively, and with an arbitrary orientation with respect to the lattice. However, we have here a situation similar to that of ordinary space groups, where the arbitrariness of the origin in space is not an obstacle to fixing this origin in a convenient way. In the same way, in the SpSG formalism, the arbitrariness of the global orientation of the spin system with respect to the lattice should not be an obstacle to choosing and fixing a convenient reference frame for the spin system with respect to the lattice. In our view, in most cases, it is convenient to choose this frame equal to that for the space operations. In this work, we will then express the operations *U* and *R* of any operation 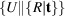 in a common reference system defined by the conventional unit cell and the crystallographic axes which are normally used for the description of the space operations. This does not imply any loss of generality, as an arbitrary global orientation of the *U* operations with respect to the crystallographic axes can always be introduced if desired, when describing these operations in the chosen reference frame.

In addition, in most cases, magnetic anisotropy cannot be fully ignored and spins have a very specific relative orientation with respect to the crystallographic axes. Even with a hypothetical null SOC and the energy being independent of the relative orientation of spin and space operations, magnetic crystal tensor properties are measured and quantified in a reference frame locked to the crystal structure, and therefore their symmetry-adapted form in this reference frame depends in general on the relative orientation of the spin and space operations. Therefore, for practical reasons, when dealing with the SpSG of a specific structure, the spin operations in the SpSG will be described (locked) under the specific spin-lattice orientation observed in the structure. As shown below, this allows a consistent comparison of the SpSG and MSG symmetries that can be assigned to the structure, and their corresponding constraints.

### 2.3. Spin point groups

For the symmetry properties of crystal tensors, only the SpPG is relevant. This is formed by the pairs of point-group operations 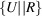 present in the SpSG operations. The subgroup of operations 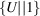 form the *spin-only* point group P_SO_. Similarly to equation (7), the full SpPG can be decomposed in a direct product of a so-called ‘non-trivial’ SpPG, P_NT_, and the spin-only point group P_SO_: 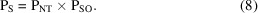
However, the similarity with equation (7) may be misleading because, as discussed above, G_NT_ may have operations of type 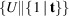, with 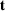 not being a lattice translation. These operations form the so-called spin-translation group G_ST_, and their point-group operations 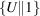 will belong to P_SO_. Hence, the SpPGs P_NT_ and P_SO_ do not necessarily coincide with the point groups separately associated with G_NT_ and G_SO_, P_SO_ being in general a supergroup of the point group associated with G_SO_. This means that while there are only two possible *spin-only* space groups G_SO_, associated with collinear and coplanar structures, the number of possible *spin-only* point groups P_SO_ does not have this restriction and may also be relevant for non-coplanar SpSGs.

The spin-only subgroup P_SO_ in equation (8) can then generally be decomposed in the direct product of two subgroups: 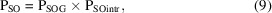
where P_SOintr_ is the intrinsic (or trivial) point group 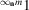 or 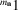 present in collinear and coplanar SpSGs, and P_SOG_ is the spin-only point group that may be present in the non-trivial G_NT_. The additional P_SOG_ must be considered only in the case that the *klassengleich* index 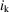 of the subgroup L_0_ with respect to G_0_, mentioned above, is larger than one, such that the translation lattice of L_0_ is a sublattice of the lattice in G_0_. For 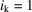 and a non-coplanar SpSG, the SpPG is directly a non-trivial SpPG, and no spin-only subgroup must be considered. Liu *et al.* (2022 ▸) enumerated collinear and coplanar SpPGs by restricting P_SOG_ to be the identity. These groups would be valid if 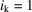 and could be useful for local symmetry studies even if 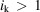. In our study of macroscopic properties, however, P_SOG_ must necessarily be included.

The fact that, in contrast to SpSGs, the spin-only point subgroups P_SO_ are not limited to two, and are not generally trivial, means that the term ‘non-trivial’ assigned to the point group P_NT_ in equation (8) is somehow ill-founded. We however stick to this terminology. A derivation of the possible non-equivalent non-trivial SpPGs P_NT_ in equation (8) was done by Litvin (1977 ▸), and a total of 598 were enumerated. This derivation was done taking into account that the point-group operations *U* can only be crystallographic.

The structure of the SpPG described in equation (8) allows the derivation of the symmetry-adapted form of any tensor in a stepwise form, considering first the constraints caused by the non-trivial group P_NT_ and then adding those coming from P_SO_. In many cases, P_NT_ can be chosen to coincide with the actual MPG of the structure, and P_SO_ is only the intrinsic spin-only subgroup, associated with the collinearity or the coplanarity of the structure (see Section 3). In such cases, the SpPG form of the tensor can then be obtained by just adding the constraints due to P_SOintr_ to those under the MPG of the structure.

## 3. Relation between spin and magnetic groups

By definition, the SpSG of a magnetic structure does not depend on the global orientation of the spin system with respect to the lattice. However, if spin and space operations are described in the same reference frame, the subgroup of operations 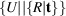 that fulfill 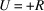 or 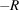 constitute according to equations (2) and (6) an MSG, which proves to be the MSG of the structure if (and only if) the SpSG is being described under the specific relative spin-lattice orientation observed in the structure. Only under this condition do the SpSG and the actual MSG of the magnetic structure have a group–subgroup relation. Conversely, the same SpSG can have different MSGs as subgroups depending on the chosen orientation of the spin operations *U* with respect to the lattice, and as a consequence, the same SpSG can be associated with magnetic structures that have very different MSGs.

Therefore, the application of the SpSG symmetry on a magnetic structure and its comparison with its MSG requires a specific orientation of the spin operations *U* to be fixed with respect to the lattice, which must be consistent with the spin-lattice orientation observed in the structure. In the following, if no indication to the contrary is given, the SpSG and the SpPG of a magnetic structure will be described fulfilling this condition. In this way, the stronger symmetry constraints on the tensors under its SpPG can be compared with those expected under the MSG, when SOC effects are taken into account. This is consistent with the fact that in experimental magnetic structures the spins have a specific global orientation (and domain-related ones) with respect to the lattice, as magnetic anisotropy is generally present in some form. In axial symmetric or pseudo-symmetric systems the spin orientation on the basal plane often remains undetermined, but in most cases, it is an experimental problem rather than a physical one.

We distinguish two types of experimental magnetic structures, depending on their SpSG–MSG group–subgroup relation, namely structures with minimal SpSG and structures with non-minimal SpSG.

(i) *Magnetic structures with minimal SpSG*. In these structures both their SpSG and their MSG have the same space operations. A majority of the observed commensurate magnetic structures enter into this group. A necessary condition for this to happen is that the *klassengleich* index 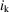 of the SpSG, described in Section 2.2, is either 1 or 2, as if 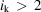 the SpSG must include some operations of type 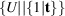 with 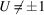, whose space operations (namely translations) cannot be present in the MSG. Thus, 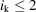 is required to ensure that the spin-only point group of the SpSG of these structures is limited to P_SOintr_ plus the additional time-reversal operation, 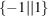, in the case of 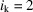.

The only difference between the SpSG and the MSG of a structure with minimal SpSG is the intrinsic spin-only subgroup in the case of collinear and coplanar structures, while in non-coplanar structures, both groups fully coincide. Therefore, for non-coplanar structures of this type, SpSG symmetry considerations do not add any additional constraint on their material tensors. However, in collinear and coplanar structures with minimal SpSG, the spin-only subgroup makes a difference. Their SpSG can be expressed as the direct product of the actual MSG of the structure with the corresponding collinear or coplanar spin-only group, and the corresponding point groups will satisfy similar relations, namely: 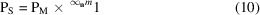

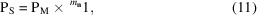
where P_S_ and P_M_ are the SpPG and MPG of the structure and 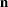 defines the orientation of the collinear or coplanar arrangement, as discussed above. As shown below with some examples, this implies that the symmetry-adapted form of any spin-related tensor for these structures under the SpPG can be simply derived taking the tensor form under the MPG, obtained by applying the usual known rules, as can be obtained for instance in *MTENSOR* (Gallego *et al.*, 2019 ▸), and then introducing the additional constraints resulting from the extra symmetry represented by P_SOintr_.

Magnetic structures with minimal SpSG can be easily identified by comparing their MSG label in the Opechowski–Guccione (OG) notation (Campbell *et al.*, 2022 ▸) with the four-index label of the non-trivial subgroup of their SpSG in the notation of Chen *et al.* (2024 ▸). The space group, denoted G_0_ in Section 2.2, formed by the space operations 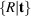 of the non-trivial SpSG, must coincide with the space group associated with the MSG, which is formed by all its operations, disregarding the inclusion or not of time reversal. This latter space group is called the family space group F of the MSG (Litvin, 2013 ▸; Campbell *et al.*, 2022 ▸). Therefore, magnetic structures with minimal SpSG fulfill 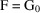. The space-group type of F is given by the first number of the numerical label of the MSG in the OG notation (using the space-group numerical indices of the *International tables of crystallography*), while the second number in the four-index notation of Chen *et al.* (2024 ▸) corresponds to G_0_. If these two numbers coincide, and 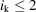, G_0_ and F necessarily coincide. The two space groups are not only of the same type, but because of the restriction on the 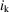 value, they must be the same space group, and the structure has a minimal SpSG.

From the approximately 2000 entries of commensurate magnetic structures in the MAGNDATA database (Gallego *et al.*, 2016 ▸) about 1500 have minimal SpSGs. We can therefore infer that in approximately 75% of the cases the differences in the symmetry-adapted tensor forms when considering MPG or SpPG symmetries are limited to the additional constraints coming from P_SOintr_ in the case of collinear and coplanar structures.

(ii) *Magnetic structures with non-minimal SpSG*. These are the structures where their SpSG includes space operations that are not present in their MSG. About 25% of the commensurate structures in MAGNDATA have non-minimal SpSGs, with their G_0_ being a strict supergroup of F: 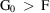. The *klassengleich* index 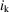 of the non-trivial SpSG being larger than 2 is a sufficient condition for this strict group–subgroup relation to be satisfied, but it can also happen for 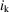 = 1 or 2. In such structures, it is clear that the additional SpPG symmetry constraints cannot be reduced to those coming just from P_SOintr_, because the point group of the non-trivial SpSG will be a strict supergroup of the MPG. By definition, the space-group operations in G_0_ must keep the positional crystal structure invariant. Therefore, G_0_ can only be a strict supergroup of F if the magnetic ordering is such that the space group F associated with the MSG loses some of the space-group operations of the paramagnetic phase. If we call G_P_ the space group of the paramagnetic phase, then in general for this second type of commensurate magnetic structures 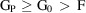. Whether or not 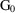 is a strict subgroup of G_P_ makes no difference when it comes to reducing crystal tensors. We will see examples of both situations later.

It will be shown below in detail that there are tensors, such as those involving only space degrees of freedom, or those involving orbital degrees of freedom, where only the space parts *R* of the operations of the SpPG are relevant for their transformation properties. The symmetry-adapted form of these tensors under an SpPG can therefore be derived considering only the space operations in the SpPG, as done in ordinary MPGs. In the case of orbital-related tensors, one has also to consider if the operation includes time reversal or not, but the specific spin operation *U* is irrelevant. It is therefore convenient to define, for a given SpPG, an *auxiliary* ordinary MPG that we denote as the *effective* MPG, MPG_eff_, which can be used instead of the full SpPG to derive the symmetry-adapted form of these non-magnetic tensors or orbital-related tensors. The MPG_eff_ is constructed by taking the space part *R* of each 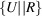 operation of the SpPG, without time reversal (*R*) or with time reversal (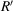), depending on whether 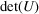 is 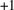 or 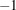, respectively. The MPG_eff_ is, in general, a supergroup of the actual MPG of the structure. The symmetry constraints under the SpPG on the mentioned type of tensors can then be obtained by considering this MPG_eff_ instead of the real MPG, when applying the well known rules for MPGs (Gallego *et al.*, 2019 ▸). The MPG_eff_ of collinear and coplanar structures is just the gray point group resulting from adding the time-reversal operation to the point group of the space group G_0_ associated with the SpSG. This is because in both collinear and coplanar structures their spin-only group, P_SOintr_, includes at least an operation 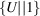 with 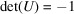, and therefore the corresponding MPG_eff_ contains the time-reversal operation. Thus, if P_0_ is the point group of G_0_, the corresponding 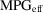 can be expressed as 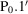. Only if the structure has a non-minimal SpSG will this gray point group 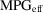 include point-group operations *R* that are not present in its actual MPG.

## 4. Tensor transformations under spin point-group operations

Given a physical property represented by a tensor *A*, the symmetry restrictions that a SpSG forces on *A* can be found by knowing the way in which the operations 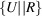 of the SpPG transform that tensor. According to the Neumann principle generalized to SpSGs, the operations of the SpPG on the tensor must leave it invariant, *i.e.* we can symbolically write 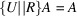.

The specific action of an operation 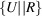 depends greatly on the nature of the tensor considered. This complexity, which is already found when trying to reduce tensors according to the MPGs (Birss, 1963 ▸; Grimmer, 1993 ▸; Grimmer, 1994 ▸; Kleiner, 1966 ▸; Cracknell, 1973 ▸; Shtrikman & Thomas, 1965 ▸; Kopský, 2015 ▸), is higher when dealing with the SpPGs. We will begin our discussion by considering the action of 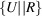 on various tensors of rank 1, starting with examples where such action is simple and direct. These cases are those in which only the *R* part or only the *U* part is involved in the transformation.

### 4.1. Pure-lattice and pure-spin vectors

Pure-lattice and pure-spin vectors are tensors of rank 1 whose transformations only involve either the space part 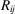 or the spin part 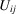 (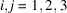) of the SpPG transformation. An example of a pure-lattice vector is the electric polarization 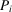, and an example of a pure-spin vector is the spin component of the magnetization 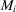. The transformations in these cases have the familiar forms:
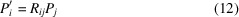

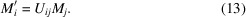
Note that, given the definition of *U* in Section 2, it is not necessary in equation (13) to multiply the right-hand side by the determinant of *U* even though 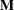 is an axial vector.

The possible orbital contribution to the magnetization is not included in equation (13) and will be ignored for the moment. This will be incorporated later in our treatment.

The two quantities 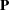 and 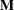 are prototypes of two of the four basic ferroic effects. These four effects are rank-1 tensors which differ from each other by their specific transformations under the space inversion 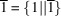 and time reversal 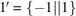. They are key to analyzing the action of 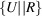 on the various tensor quantities, and we will assign them different labels (V, *e*V, M, T), which specify the four different behaviors shown in Table 1 ▸.

Thus, with reference to this table, we say that 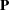 is a tensor of type V (polar Vector) and 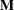 is a tensor of type M (axial Magnetic vector). The prototypes of the other two basic effects are the moment of the polarization 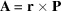 (*e*V, axial pure-lattice vector) and the moment of the magnetization or Toroidic moment 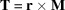 (T, polar mixed vector).

The transformation of a vector of type *e*V under 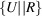 is also simple, 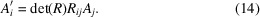
The simplicity of equation (14) comes from the fact that both 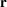 and 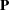 are pure-lattice vectors, and in their transformation only the space part *R* of the operation intervenes. This is, however, not the case for a tensor of type T, which involves both space and spin operations *R* and *U*, and whose analysis will be postponed until after the discussion of the transformations of the magnetoelectric tensor.

### 4.2. The magnetoelectric tensor

The magnetoelectric effect is described by a tensor of rank 2 that describes either the magnetization induced by an applied electric field 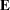 (inverse effect, 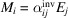) or the polarization induced by an applied magnetic field 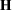 (direct effect, 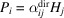).

Since 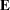 is a pure-lattice vector and 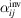 must transform as the product 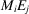, we easily obtain the transformation law of the inverse effect, 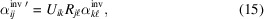
*i.e.* tensor 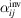 is a tensor of rank 2, whose transformation mode involves *R* and *U*. We say that 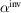 is a tensor of type MV.

The direct effect can be analyzed similarly. Thermodynamic arguments indicate (Nye, 1985 ▸) that the tensor of the direct effect is equal to the transpose of the tensor of the inverse effect 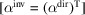, so taking the transpose of equation (15) we have 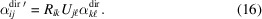
In the following we will use the symbols 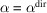 and 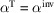 for the direct and inverse effects, respectively.

### 4.3. The toroidic moment

The transformation law for the toroidic moment 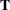 can be deduced by noting that this quantity transforms just like the antisymmetric part of the magnetoelectric tensor (direct or inverse effect) (Spaldin *et al.*, 2008 ▸). This can be deduced by noticing that the quantities 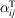 (we take the inverse effect as an example) transform as the product 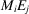, so that 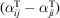 will transform as 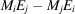. Since the electric field transforms as the position vector 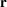, then 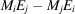 will transform as the *k* component of the vector 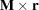, which corresponds to the association 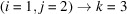 and circular permutations.

The components 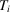 can therefore be assimilated in the quantities 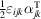 from the point of view of their transformation laws, where 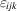 is the Levi-Civita symbol. We can say that 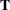 is a quantity of type {MV} (or {VM}), the curly brackets denoting the antisymmetric part. If we define a tensor 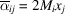 (where 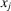 are the components of 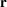), then we directly have 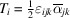. Writing this tensor as a sum of a symmetric part 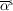 and an antisymmetric part 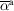, *i.e.*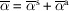, with 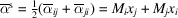 and 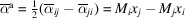, we will have from equation (15) 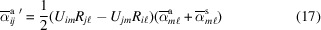

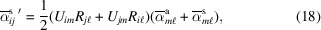
from which we can deduce the transformation law for 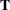. It is interesting to note that equations (17) and (18) indicate that the transformations for 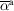 and 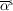 are, in general, coupled. In other words, these transformations cannot be written in the usual form 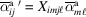 or 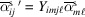 (with *X*, *Y* suitable transformation matrices), because in the right-hand sides of equations (17) and (18) there are also contributions dependent on 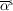 and 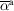, respectively. This means that neither 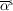 nor 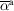 (and thus the toroidic moment) are true tensors for SpPG transformations. From equations (17) or (18) it can be deduced that 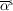 and 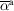 become uncoupled if 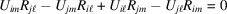
and 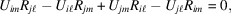
*i.e.*
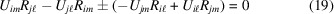
which, in general, is not satisfied. A special case occurs for the MPG operations, in which 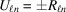. When this condition is met, it can be easily seen that equation (19) does certainly hold.

Consequently, to obtain the symmetry-adapted form of 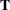, we must first consider the symmetry invariance of a tensor 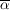 which transforms similarly to the magnetoelectric tensor, using equation (15) or (16), and then take its antisymmetric part by means of the product of this tensor with the Levi-Civita tensor. Note that this procedure does not require the use of equations (17) and (18). Thus, the component 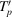 of the toroidic moment transformed by the operation 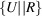 will be given by 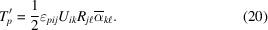
The complexity of this transformation law is a characteristic of SpPGs and leads to more laborious tensor symmetry reductions than those for MPGs.

### 4.4. Equilibrium properties

Once the transformation properties of the four basic ferroic effects have been deduced, we can obtain the corresponding transformations for the different equilibrium properties through their constitutive equations. They can be described in each case by an appropriate combination of the labels V and M, which accounts for the intrinsic symmetry of the tensor. These combinations constitute symbols that generalize the so-called Jahn symbols (Gallego *et al.*, 2019 ▸; Jahn, 1949 ▸) used with the MPGs.

Table 2 ▸ lists a selection of equilibrium properties, the constitutive equation, the Jahn symbols for the MPGs and SpPGs, and an outline of the transformation law in the case of the SpPGs. The table only lists tensors of properties where spin magnetism is involved, and therefore their transformation rules have to be modified when considering SpPG symmetry. In the case of pure-lattice tensors, the known transformation rules for ordinary space-group operations are still in place, as they only involve space operations *R*. Hence, in tensors such as electric polarization, dielectric susceptibility or piezoelectric tensor, a difference between the constraints when considering MPG and SpPG symmetry can only occur in structures with a non-minimal SpSG, where the space group G_0_ associated with the SpSG is a supergroup of the family group F of its MSG (see Section 3). The calculation of the symmetry-adapted form of these tensors under the SpPG can be obtained by applying the well known transformation rules for MPGs under the symmetry given by MPG_eff_, which was defined in Section 3.

Apart from the magnetization, there is in Table 2 ▸ one case (magnetic susceptibility) where the transformation includes only the *U* part, in which the invariance against 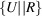 is written in the simple form:
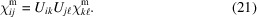
In all other examples, both *R*’s and *U*’s are involved in various combinations. Particularly complicated are the tensor transformations whose symbol includes {MV}.

An extension of Table 2 ▸, with a more comprehensive list of material properties, is given in the supporting information (Table S1).

As an example of how to find the symmetry-adapted shape of a given tensor, we choose the electrotoroidic effect 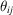 (type {MV}V). 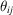 is reduced in a two-step process. First we take a type MVV rank-3 tensor, 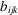, and reduce it. Then, we contract the first two indices by means of the product with 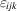. More explicitly, first we will find the 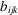 tensor invariant under all operations 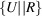 by requiring 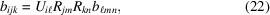
and, afterwards, we will take the antisymmetric part of 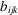 with respect to the first two indices in the form 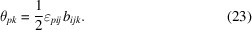
The additional symmetries indicated in Table 2 ▸ by the square brackets are easy to handle. For example, to reduce the piezotoroidic tensor 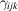 (direct effect) that transforms according to {MV}[V^2^], we will first take a type MVVV auxiliary tensor of rank 4 and require its invariance under the SpPG, *i.e.*
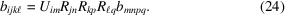
Now, once equation (24) is solved, tensor 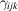 is obtained by means of the expression 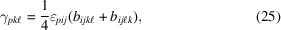
which takes out the antisymmetric part of 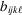 in the first two indices and symmetrizes the final tensor in the *k* and 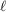 indices.

We end this section by noting that the Jahn symbols in Table 2 ▸ can be used not only to derive the symmetry restrictions of the tensors under a given SpPG, but also to obtain the relation between tensors corresponding to two structures with the same SpPG, differing only in a global spin rotation. Thus, if this rotation (proper or improper) is described by a matrix *P*, the new tensor is obtained from the old one after substituting *U* by *P* and taking a rotation *R* equal to the identity, 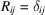, in the last column of Table 2 ▸. For example, in the case of the magnetization 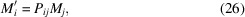
where 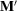 is the magnetization of the structure with the spins rotated. Similarly, the magnetic susceptibility of the rotated spin structure 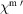 will be 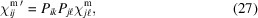
and in the case of the inverse magnetoelectric tensor we will have 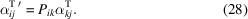
This is of interest, for example, for relating the tensors of two collinear (or coplanar) structures with different orientations of the spin direction (or of the spin plane) with respect to the lattice. Note, however, that their scope is wider and they can be used more generally, even with non-coplanar structures.

In this respect it is interesting to point out that one could alternatively define the symmetry-adapted form of the tensors under a SpPG using two different reference frames for spin and lattice variables, so that the spin-related indices of the tensor refer to a spin reference system independent of the one used for the lattice. This approach permits a general description for the tensors under the SpPG symmetry. For example, the magnetoelectric tensor can be defined as a tensor 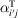 with unprimed lattice indices and primed indices referring to the spin space. Thus, the physical meaning of this coefficient is that an electric field along *j* in the lattice induces a magnetization along 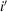, the direction 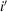 being defined with respect to the reference frame of the spins, which can be chosen totally independent of the lattice. We will return to this point later, when we analyze some examples.

### 4.5. Equilibrium properties related to orbital degrees of freedom

The Jahn symbol of all tensors in Table 2 ▸ contains the letter ‘M’, as it corresponds to magnetic tensor properties resulting from the electronic spins. But, in general, all these tensors may also have a contribution of orbital origin. As discussed in Section 2.1, in contrast to the atomic spins, orbital magnetic moments are locked to the lattice even in SOC-free systems, and therefore the orbital part of these tensors transforms according to the usual Jahn symbol for MPGs. For instance, upon an operation 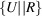 of the SpPG, the magnetization 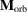 of the orbital origin is transformed as a magnetic axial vector according to the space operation *R*, incorporating the possible time reversal if 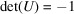 (Watanabe *et al.*, 2024 ▸). Thus, we will have the counterpart of equation (13), 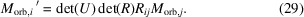
The associated Jahn symbol is *ae*V, as in an ordinary MPG. Note, however, that here the MPG to be used is MPG_eff_, described in Section 3, whose elements in the 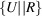 notation are of the form 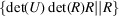.

Orbital contributions of properties listed in Table 2 ▸ are thus transformed differently from their spin contributions. For example, the magnetoelectric tensor (inverse effect) has an orbital component 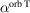 whose transformation law corresponds to the Jahn symbol *ae*V^2^. This means that for an operation 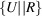 the transformation is of the form 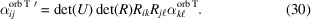
The toroidic moment also has an orbital component 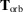. Since the operations 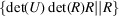 of MPG_eff_ verify equation (19), the transformation law is simpler here than in the case of the spin component. For the orbital contribution, we have decoupled the transformations of the symmetric and antisymmetric parts of the magnetoelectric tensor, which gives rise to the simple result:
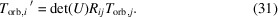
As a last example we take the orbital part of the magnetic susceptibility, which transforms as 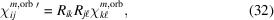
*i.e.* in the same way as in a non-magnetic crystal.

Therefore, in general, the symmetry-adapted form of the tensors of orbital origin can be simply derived using the transformation rules for the MPG_eff_. The tensors for the full properties are then the sum of the tensors for the spin and orbital contributions. This has important simple consequences because, as shown in Section 3, the MPG_eff_ of all collinear and coplanar structures are gray. This implies that the orbital contribution to all tensors that are odd under time reversal (*i.e.* ‘*a*’ present in the Jahn symbol) is necessarily null in collinear and coplanar structures, if SpSG symmetry is valid. On the other hand, for tensors that are even under time reversal (*i.e.* ‘*a*’ not present in the Jahn symbol), the collinearity or coplanarity of the structure does not introduce any specific restriction to their orbital contributions. Finally, it should also be noted that the tensors accounting for the orbital contributions under the symmetry constraints of a SpPG are independent of the global orientation of the spin arrangement.

Table S1 in the supporting information also shows separately the transformation rules for the orbital and spin contributions in a selection of equilibrium properties.

### 4.6. Constraints on equilibrium tensors of collinear and coplanar magnetic structures

As indicated by equation (8), any SpPG is the direct product of a non-trivial part and a spin-only point group. Collinear and coplanar structures are characterized by the fact that they always possess a certain minimum symmetry, P_SOintr_, in their spin-only point group P_SO_. This symmetry alone produces certain general restrictions on some tensor properties, which can be derived separately.

In collinear materials the spin operations *U* of P_SOintr_ form the continuous group 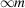, and in the coplanar case the group *m*. In order to derive the tensor constraints on a general basis, and following the usual convention, we take the *z* axis parallel to the spins in the case of collinear groups, and in the case of coplanar groups the plane of symmetry is taken perpendicular to *z*. Hence, the generators of the two P_SO_ are 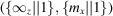 and 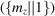, respectively. For each specific structure, the resulting tensor constraints derived for this generic *z* direction will then have to be translated to the actual collinear or coplanar orientation with respect to the lattice, which is present in the structure. These operations strongly constrain the form of some tensors, as shown in Table 3 ▸. The table only lists tensors for spin magnetism contributions. The constraints resulting from collinearity or coplanarity in the case of tensor contributions of orbital origin were discussed in the previous section, where they were reduced to the simple rule that tensors odd for time reversal are null, while for even ones they do not imply any specific restriction. This means that time-odd tensors in collinear and coplanar structures under SpSG symmetry can only have contributions of spin origin. For pure-lattice tensors, where only space operations are involved, obviously collinearity or coplanarity do not introduce any specific restriction, and are not included in the table either.

The constraints described in Table 3 ▸, resulting from the collinearity or coplanarity of the structure, *i.e.* from P_SOintr_, must be added to the symmetry-adapted form of the tensor deduced from the non-trivial subgroup of the SpPG, and the non-intrinsic spin-only group (if it exists). In the case of structures with minimal SpSG (see Section 3), it is sufficient to add the collinear or coplanar constraints described in the table to the symmetry-adapted form of the tensor for the actual MPG of the structure.

When dealing with properties where the spin contribution to the toroidic moment is involved, Table 3 ▸ does not directly show the constraints due to collinearity or coplanarity. Instead, it indicates the restrictions on the tensors out of which these quantities are constructed by antisymmetrizing two of the indices. For example, for the inverse electrotoroidic effect 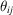 (type {VM}V), the form corresponding to a 3-index tensor of type VMV, 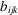, is indicated. It is on this *extended* tensor that the rest of the SpPG constraints must be applied when deducing the final form of the property in question. This issue is due to the fact that 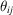 is not really a genuine tensor for SpPG transformations (since 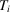 is not, see Section 4.3).

In the supporting information we have extended Table 3 ▸ with more properties, and also explicitly list the restrictions on orbital contributions where applicable (Table S2).

### 4.7. Transport phenomena

For non-equilibrium transport properties, it is the Onsager theorem, and not the constitutive relationships, that indicates how these tensors transform under the time-reversal operation (Butzal & Birss, 1982 ▸; Eremenko *et al.*, 1992 ▸; Grimmer, 1994 ▸; Shtrikman & Thomas, 1965 ▸). For example, it can be shown from the Onsager theorem that the electrical resistivity ρ, which relates electric field 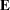 and current density 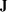 (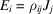), is transformed by time reversal in the form: 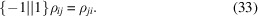
This expression allows the definition of a symmetric part 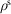 and an antisymmetric part 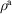 (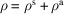) (Grimmer, 2017 ▸) that are even and odd for time reversal, *i.e.*
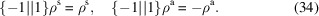
Therefore, since the electric field 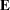 is a vector of type V, we deduce that 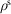 must be a tensor of type [V^2^]. As for 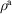, it should be noted that although in principle the *U* part of 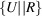 affects neither the electric field nor the current density, the second of equations (34) implies that there must be a sign change in the transformation if the time reversal is included in the 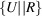 operation, *i.e.* if *U* is improper.

Thus, for the symmetric part we have 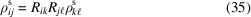
and for the antisymmetric part 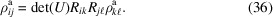
In other words, 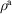 is an antisymmetric magnetic tensor, whose Jahn symbol is *a*{V^2^}, just as with ordinary MPGs. 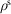 accounts for the ordinary electric resistivity, whereas 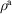 is responsible for the anomalous (or spontaneous) Hall effect.

Similar to the orbital components of the equilibrium properties, the transformations of 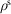 and 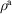 by the SpPG are formally identical to those of an MPG, and, therefore, the symmetry-adapted form of the tensors can be obtained just with the methods employed for MPGs, applied to the MPG_eff_ that can be associated with the SpPG.

It is interesting to note that the restrictions imposed by the SpPGs on the magnetization and 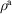 are not equivalent. This is in sharp contrast to the case of the ordinary MPGs, where it can be shown that the Jahn symbols for 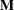 and 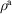 (*ae*V and *a*{V^2^}, respectively) are equivalent, in such a way that the occurrence of magnetization is closely linked to the existence of the anomalous Hall effect. However, in the framework of SpPGs, the equivalence in the transformation law is between the anomalous Hall effect and just the orbital part of the magnetization. Therefore, it can be the case of having 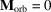 so that 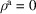 (without SOC), and yet there is a non-zero spin component of the magnetization. Thus, there are ferromagnetic systems where the anomalous Hall effect can only be a SOC effect. Conversely, antiferromagnetic (non-coplanar) structures may exhibit an anomalous Hall effect, even with the spin macroscopic magnetization being zero (Watanabe *et al.*, 2024 ▸).

The application of external magnetic fields leads to the definition of new effects that are described by tensors of ranks higher than 2. For example, keeping only terms linear in 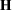, 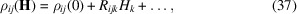
and separating symmetric and antisymmetric parts, we have two tensors, 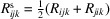 and 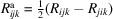, symmetric and antisymmetric in the first two indices, respectively. The symmetric part of the spin component 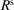 is of type [V^2^]M and accounts for the linear magnetoresistance, while the spin contribution to 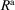 is of type *a*{V^2^}M, and is the tensor describing the ordinary Hall effect (Grimmer, 2017 ▸). The meaning of these symbols is as follows: 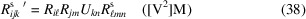
and 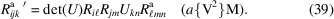
These transformations are also valid for the spin Hall resistivity tensor, 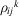, that connects the electric field with the spin current polarized in the *k* direction 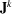 (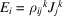) (Seemann *et al.*, 2015 ▸; Železný *et al.*, 2017 ▸). Since the relation between 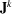 and 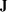 is just a term that is transformed as the spin (type M), it follows that the symmetric part of 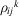 in *ij* must also transform as [V^2^]M and the antisymmetric part as *a*{V^2^}M.

Tensors 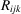 and 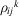 also have orbital components (orbital Hall tensor and orbital-current Hall resistivity) (Bernevig *et al.*, 2005 ▸). For example, in the case of the Hall effect, we will have for the symmetric and antisymmetric parts the transformations 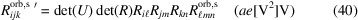
and 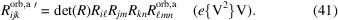
We end this section with a reference to thermoelectric tensors Seebeck β and Peltier π. The Seebeck effect relates a temperature gradient 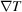 to the appearance of an electric field (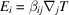), and the Peltier effect connects an electric field with a heat flux density 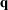 (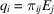). For the Seebeck and Peltier effects the Onsager relations lead to 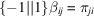 and 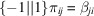 (Gallego *et al.*, 2019 ▸). It is then interesting to take the combinations 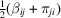 and 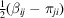, which are invariant and anti-invariant under time reversal, respectively (Grimmer, 2017 ▸). From these behaviors under 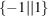, and following the same reasoning as in the case of 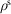 and 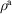, it can be deduced that 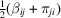 must be a V^2^ tensor and 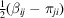 must be of *a*V^2^ type. The former is responsible for the ordinary Seebeck effect and the latter for the so-called spontaneous Nernst effect.

Table 4 ▸ contains a summary of the transformation properties of the spin contributions for some transport tensors. As with the tensors discussed in the previous section, in the case of those having Jahn symbols without the letter ‘M’, the additional constraints resulting from the SpPG can be simply obtained by comparing their symmetry-adapted forms under MPG_eff_ with those under the actual MPG of the structure. A table including more transport properties together with the separation of their orbital and spin parts, where applicable, is shown in the supporting information (Table S3).

### 4.8. Constraints on transport tensors of collinear and coplanar magnetic structures

Similarly to equilibrium tensors, the minimal intrinsic spin-only subgroup of collinear and coplanar structures can impose important restrictions on tensors describing transport properties. A compilation of these restrictions for the spin contributions of some properties is shown in Table 5 ▸. In the supporting information we show further transport properties and separate the constraints for the orbital and spin parts, where relevant (Table S4). In some cases, the constraints are very important. For example, the antisymmetric part of the resistivity is forbidden in collinear and coplanar structures, and therefore the anomalous Hall effect can only be non-relativistic in non-coplanar magnetic structures (Taguchi *et al.*, 2001 ▸; Nagaosa *et al.*, 2010 ▸). The same happens for the spontaneous Nernst and Ettingshausen effects. Similarly, the spin Hall resistivity tensor is highly restricted in collinear and coplanar materials, with the antisymmetric part of the tensor totally vanishing in the case of collinear structures (Zhang *et al.*, 2018 ▸). In contrast, the orbital contribution of the antisymmetric part of the Hall effect tensor (orbital part of the ordinary Hall effect, see Table S4 in the supporting information) is not restricted by the collinearity or coplanarity, and in fact that property can exist in materials of any symmetry.

### 4.9. Optical properties

The optical behavior of a material is based on the properties of its dielectric permittivity tensor at high frequencies 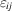, as well as on the changes that this tensor undergoes when the material is subjected to external influences (magnetic fields, electric fields, stress…). As we have pointed out in our study of equilibrium properties, the permittivity tensor is of type [V^2^] for static electric fields. However, at optical frequencies the material response is not in equilibrium. It can be shown that Onsager’s relations give rise to an expression similar to equation (33) for the action of time reversal on the optical dielectric tensor (Eremenko *et al.*, 1992 ▸), *i.e.*
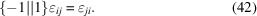
Following the same reasoning as for the resistivity, the separation into symmetric and antisymmetric parts, 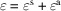, even and odd for time reversal, gives rise to the following Jahn symbols: [V^2^] for 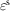 and *a*{V^2^} for 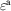. The symmetric term describes the index ellipsoid and the antisymmetric part the spontaneous Faraday effect.

The variation of 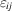 due to the space dispersion (dependence with the light wavevector 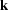), the application of an electric field and the application of a magnetic field can be written, respectively, as 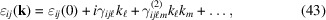

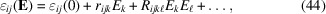

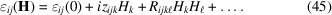
Again, if we separate 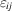 into symmetric and antisymmetric parts, and take into account the properties of transformation of 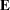, 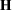 and 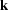 (the latter being a rank-1 tensor that changes sign both under inversion 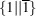 and under time reversal 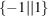), we can easily deduce the Jahn symbols of the various tensors involved. A summary of some of the effects up to rank 3 is given in Table S5 of the supporting information. Table 6 ▸ shows just the case of the spin contribution to the Faraday effect tensors (symmetric and antisymmetric parts), where the Jahn symbols for SpPGs are different from those for MPGs.

If the medium is non-dissipative it can be shown that 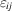 must be Hermitian (Landau & Lifshitz, 1960 ▸), *i.e.*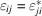. If this situation arises, it can be easily shown that the symmetric and antisymmetric parts of the various tensors must be real or purely imaginary. So, for example, for the Pockels tensor *r*, defined in equation (44), the symmetric part 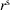

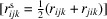
 is a real tensor and the antisymmetric part 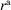

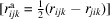
 is purely imaginary. The presence of *i* in equations (43) and (45) makes the antisymmetric part of 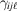 and 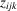 (natural optical activity and ordinary Faraday effect) real.

### 4.10. Constraints on optical tensors of collinear and coplanar magnetic structures

As in the preceding cases, collinearity and coplanarity also impose restrictions on tensors for optical properties, as is shown in Table S6 of the supporting information. Since some optical tensors share the same Jahn symbol with some of the transport tensors listed in Tables 4 ▸ and S3, their constraints can also be deduced from those tables. For example, the spontaneous Faraday effect, the spin contribution of the ordinary Faraday effect and the spin contribution of the magneto-optic Kerr effect tensors have the same shape as the antisymmetric part of the resistivity, the antisymmetric part of the spin Hall resistivity and the symmetric part of the spin Hall tensors, respectively. Consequently, P_SOintr_ already restricts greatly the form of these tensors both in collinear and coplanar structures. Other properties that can be readily shown to vanish for collinear and coplanar structures under SpSG symmetry are the spontaneous gyrotropic birefringence and the antisymmetric part of the Pockels effect (Table S6). Table 7 ▸ shows as an example the restrictions for the spin contributions to the Faraday tensors.

In the supporting information we complete our study of crystal tensors, giving an account of the transformation properties (Section S2) and constraints (Section S3) given by the SpPGs on some non-linear optical (NLO) properties. The main conclusion is that such tensors can be studied on the basis of the MPG_eff_ exclusively.

## 5. Examples

In the following we will present several examples of experimental magnetic structures with non-coplanar, coplanar and collinear ordering for which we will obtain the symmetry-adapted tensor forms for some selected properties. All the examples have been retrieved from the MAGNDATA database of the Bilbao Crystallographic Server (Gallego *et al.*, 2016 ▸).

We will introduce examples of the two types of magnetic structures that can be distinguished regarding the relation of their MSG and SpSG, which were discussed in Section 3. These two types are, on the one hand, the structures with a minimal SpSG, where the SpPG only differs from the MPG by the inclusion of the intrinsic spin-only subgroup P_SOintr_ (if collinear or coplanar), and the remaining ones, where the MPG is a strict subgroup of the SpPG, with the SpPG having additional space operations and/or non-trivial spin-only operations 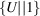. As explained in Section 3, in order to determine the relation between the MSG of a magnetic structure and its SpSG, the SpSG must be described by choosing the orientation of the spin operations with respect to the lattice, consistently with the observed structure.

### 5.1. Structures with a minimal SpSG

As has been pointed out in Section 3, a majority of the reported magnetic structures have a minimal possible SpSG with respect to their MSG, where the family group F of the MSG is equal to the space group G_0_ of the space operations 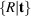 of the SpSG. Under these conditions, the MPG and the SpPG have the same set of lattice operations *R*, and the SpPG P_S_ can be written as 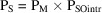, where P_M_ is the MPG of the structure and P_SOintr_ the corresponding intrinsic spin-only point group.

This has interesting consequences when it comes to obtaining the tensor reductions induced by the SpPG. Starting from the well known tensor forms under the MPG symmetry [obtained for example using the *MTENSOR* program (Gallego *et al.*, 2019 ▸)], the constraints due to the SpPG can be found by simply adding, in the case of collinear or coplanar structures, those given by P_SOintr_, which we have tabulated in previous sections. In the case of non-coplanar structures the SpPG and the MPG coincide and no additional SpPG constraint exists.

We will now examine some examples of materials that illustrate the points made above.

#### 5.1.1. Collinear DyB_4_ (entry 0.22 in MAGNDATA)

**Figure 1:**
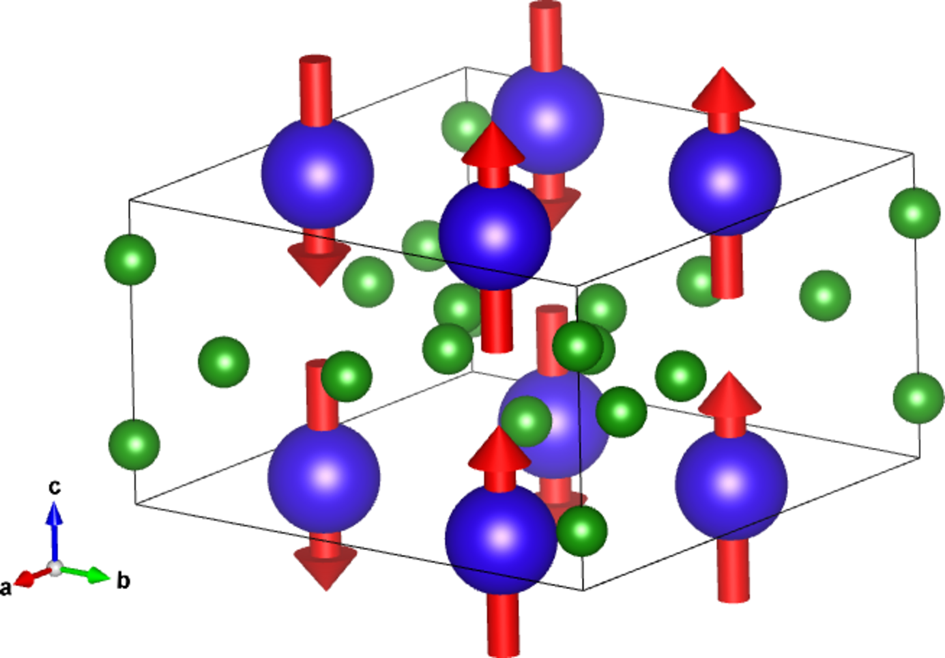
Magnetic structure of DyB_4_ below 21 K. Dy and B atoms are represented by blue and green spheres, respectively.

DyB_4_ has space group *Pbam* (No. 55) in its paramagnetic phase and below 21 K exhibits a collinear magnetic structure (Will & Schafer, 1979 ▸), with propagation vector 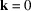 and MSG 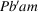 (OG No. 55.3.433), and therefore its MPG is 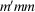. The spins are oriented along *c*. A scheme of the structure is displayed in Fig. 1 ▸. As the MSG keeps all the space operations of the parent space group *Pbam*, then the corresponding SpSG is minimal, with no additional space operation. This SpSG has been identified as 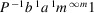 (No. 26.55.1.1) in the so-called international notation (Chen *et al.*, 2024 ▸), but one should take care that in this SpSG notation the *x* and *y* axes of the lattice have been interchanged with respect to the basis of the MSG 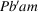. This means that, keeping the same basis as in the MSG, the non-trivial SpPG can be denoted as 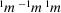, which is generated by the operations: 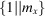, 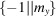 and 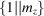. We can then write 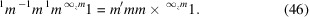
We will use equation (46) to deduce, as an example, the constraints of the magnetoelectric tensor (inverse effect) under the SpPG (see Table 2 ▸). For the MPG 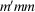 we have 
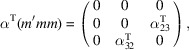
as can be easily checked. But the additional spin-only group 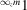 in the SpPG cancels out the elements of the first two rows (see Table 3 ▸). Therefore, the final tensor form under the SpPG symmetry is simply 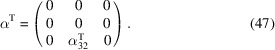
It is interesting to analyze the same case but assuming now that the spins are aligned along *a* or *b*. The counterparts of equation (46) are 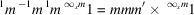
and 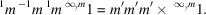
In the first case, the MPG 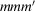 gives a tensor 
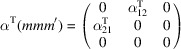
and in the second 
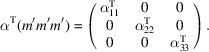
For these orientations, 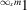 eliminates the second and third rows of 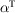, while 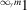 does the same with the first and third rows. Then we have under the SpPG 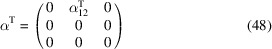
and 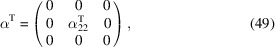
respectively.

These three results are easily interpretable. The three tensor forms for the three spin directions, equations (47)–(49), correspond to the same physical effect under the SpPG. They simply indicate that the electric-induced magnetization can only take place along the spin directions, which without SOC would be arbitrary. In contrast, independently of the direction of the spins, the electric field must be applied along a specific crystal direction, namely the *y* axis, which is the direction perpendicular to the unique mirror plane with 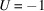 in the non-trivial SpPG. As can be seen with this example, physically equivalent tensor reductions under the same SpPG, for different orientations of the spins, can be derived starting from tensor forms under different MPGs.

Equations (48) and (49) could have been deduced from equation (47) by using equation (28), which relates the 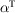 tensors in structures differing in their spin orientations. Using this procedure we easily obtain that the only surviving coefficient in equations (47)–(49) must have numerically the same value.

In the description proposed at the end of Section 4.4, the magnetoelectric tensor of this example, when described with separate spin and lattice systems, would have only a single coefficient, 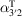, similar to equation (47). This means that an electric field along the *y* lattice direction induces a magnetization along the spin direction 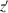, whatever this may be. Equations (47)–(49) are particular cases of this more general rule.

#### 5.1.2. Collinear MnF_2_ (entry 0.15 in MAGNDATA)

**Figure 2:**
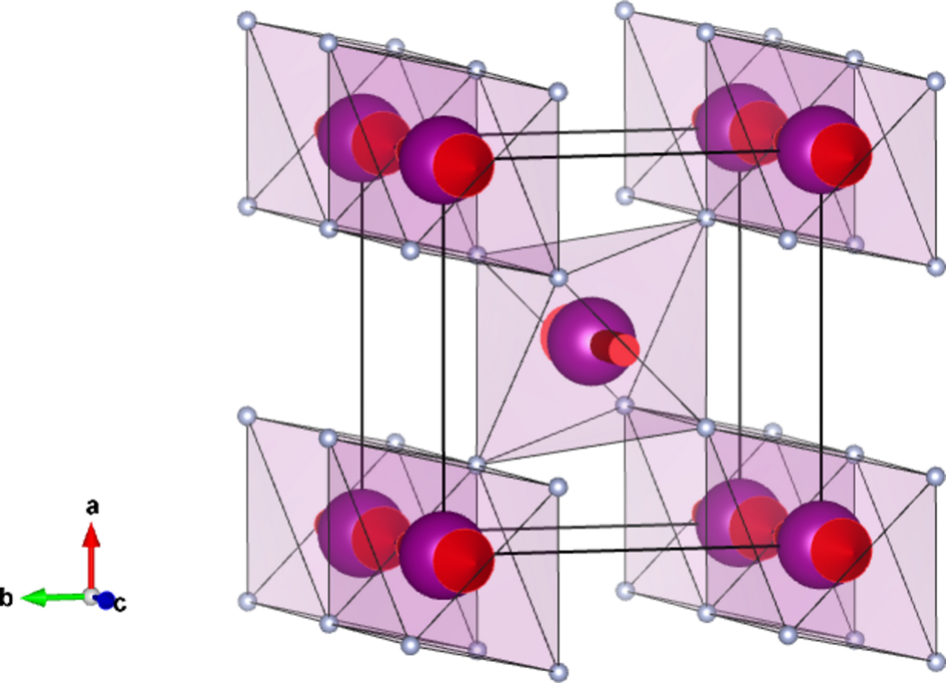
Magnetic structure of MnF_2_ showing the spins of the Mn atoms (violet spheres). F atoms are represented by small gray spheres.

MnF_2_ has space group 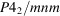 (No. 136) in the paramagnetic parent phase. Upon cooling it undergoes a transition to a collinear magnetic phase with propagation vector 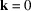 (Yamani *et al.*, 2010 ▸). The structure of the magnetic phase is shown in Fig. 2 ▸. The spins are parallel to [001], with MSG 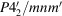 (OG No. 136.5.1156). Here again the MSG keeps all space operations of the parent space group 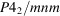, and therefore the corresponding SpSG is necessarily minimal. This SpSG is 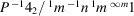 (No. 65.136.1.1) (Chen *et al.*, 2024 ▸). The corresponding SpPG, 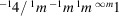, generated by the operations
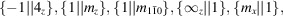
can then be related to the MPG in the form 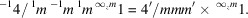
The tensor constraints according to the SpPG will then be those of the MPG plus those due to the collinearity spin-only group 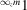.

We can take as an example the piezomagnetic tensor 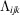 (see Table 2 ▸), which has recently been considered in connection with a discussion about the altermagnetism of this material (Bhowal & Spaldin, 2024 ▸; Radaelli, 2024 ▸). The results are obtained straightforwardly using the *MTENSOR* program and Table 3 ▸.

The constraints under the MPG give 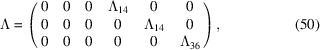
where the usual Voigt index contraction has been used for the last two indices of 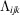. Adding the restrictions of Table 3 ▸, the only coefficient that survives is simply 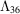. This means that the magnetization induced by stress is only along the spin direction, and it can be induced only upon application of a 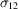 (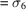) shear stress. Therefore, this is the non-relativistic piezomagnetic effect, which the system is expected to have even if SOC is negligible.

In contrast with the example of Section 5.1.1, in this case if we consider any other hypothetical spin direction for the collinear spin arrangement, the resulting MPG will lose some space operations, and therefore the SpSG will not be minimal with respect to the new MPG. Therefore, the simple method to derive the SpSG-adapted form of the tensors employed above is not possible for any other spin direction. But from equation (50) we can infer how it would be the SOC-free piezomagnetic effect in any case. Taking into account that Λ is of type M[V^2^], and following a procedure similar to the one carried out for the magnetoelectric tensor in the previous example, we easily arrive at 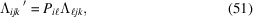
where 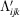 is the piezomagnetic tensor of the new structure and 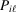 is the rotation matrix relating both spin orientations. In this case, equation (51) leaves as non-null elements only 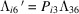 (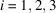). Thus, in the SOC-free limit the induced magnetization is always along the spin direction, whatever this is, but the applied stress must be a shear 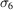 on the crystal basal plane. In the description using separate spin and lattice reference systems (end of Section 4.4) we would have here a tensor with just a single coefficient, 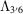, meaning that a stress 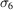 induces a magnetization along the spin direction, this being arbitrary.

#### 5.1.3. Coplanar CoSO_4_ (entry 1.519 in MAGNDATA)

**Figure 3:**
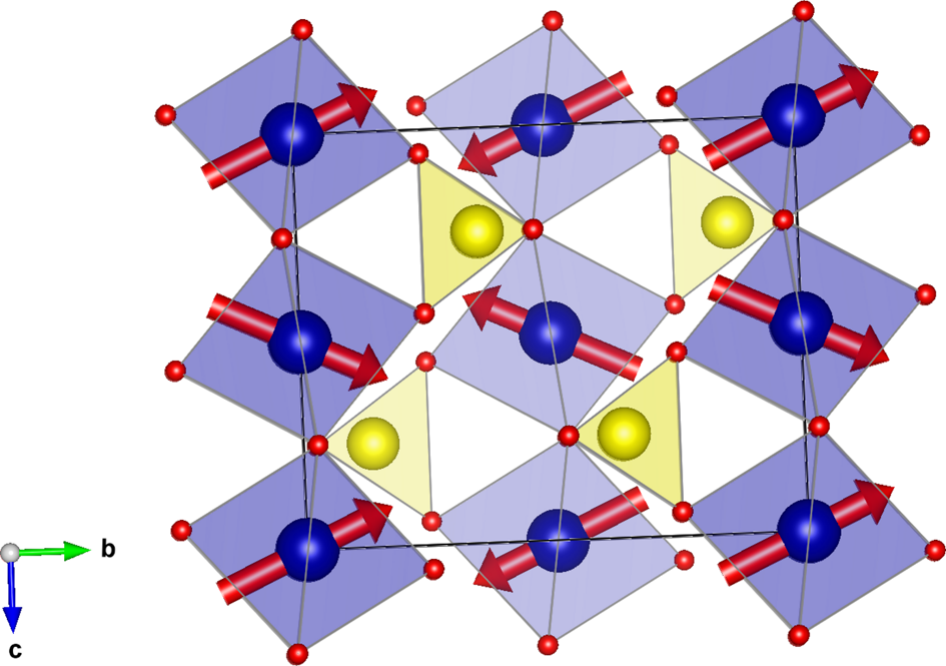
Magnetic structure of CoSO_4_ below 15.5 K showing the spins of the Co atoms (blue spheres). The O and S atoms are represented by red and yellow spheres, respectively.

CoSO_4_ has a paramagnetic phase with space group *Cmcm* (No. 63), and a magnetic phase below 15.5 K with propagation vector 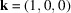 (Frazer & Brown, 1962 ▸). The material is coplanar, 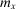 being the spin-only mirror plane (see Fig. 3 ▸). Its MSG is 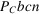 in the Belov–Neronova–Smirnova (BNS) notation, with OG numerical index 63.16.52. The non-trivial SpSG is 10.63.2.1 (Chen *et al.*, 2024 ▸). Also, in this case, despite the non-zero propagation vector, which implies the breaking of the body-centering lattice translation, all operations of the parent space group are maintained in the MSG. The lost centering translation is kept in the MSG as an antitranslation, *i.e.* a translation combined with time reversal. Thus, the MPG of the structure is 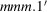, and the SpPG is necessarily minimal with respect to it. The SpPG can be written as the direct product of the MPG and the coplanar spin-only group: 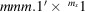. The SpPG tensor constraints can be derived, as in previous examples, by adding to the constraints of the MPG those of the 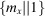 plane of 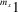.

Let us consider the spin Hall resistivity tensor as an example. The MPG restricts the antisymmetric part of that tensor to the form 
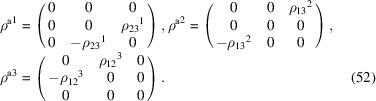
We can now add the additional SpPG constraints due to the coplanarity. According to Table 5 ▸, the SpPG only allows a non-zero 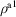 (note that the plane in P_SO_ is 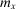 instead of 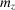) while it forces 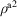 and 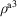 to be null. If the tensor is expressed using separate spin and lattice reference frames, the only surviving term is 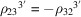, where 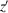 is the direction perpendicular to the spin plane, whatever its orientation with respect to the lattice.

In this case, the symmetric part of the spin resistivity is already zero under the MPG since this group contains the time-reversal operation and this part of the tensor is time-odd when considered for the MPG operations (see Table 4 ▸).

### 5.2. Structures with a non-minimal SpSG

In the examples that we will consider in this section, there are non-trivial differences between the space operations in the MPG and SpPG of the structures and/or the spin-only group P_SO_ in the SpPG is larger than P_SOintr_. In this case P_S_ cannot be written as a product 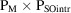. We will take two materials (and another two in Sections S5 and S6 of the supporting information) with different spin configurations, non-coplanar, coplanar and collinear, and we will review for them a certain set of selected properties, where we will compare the symmetry-adapted form of the corresponding tensors for the MPG and SpPG symmetries.

#### 5.2.1. Coplanar Mn_3_Ge (entry 0.377 in MAGNDATA)

**Figure 4:**
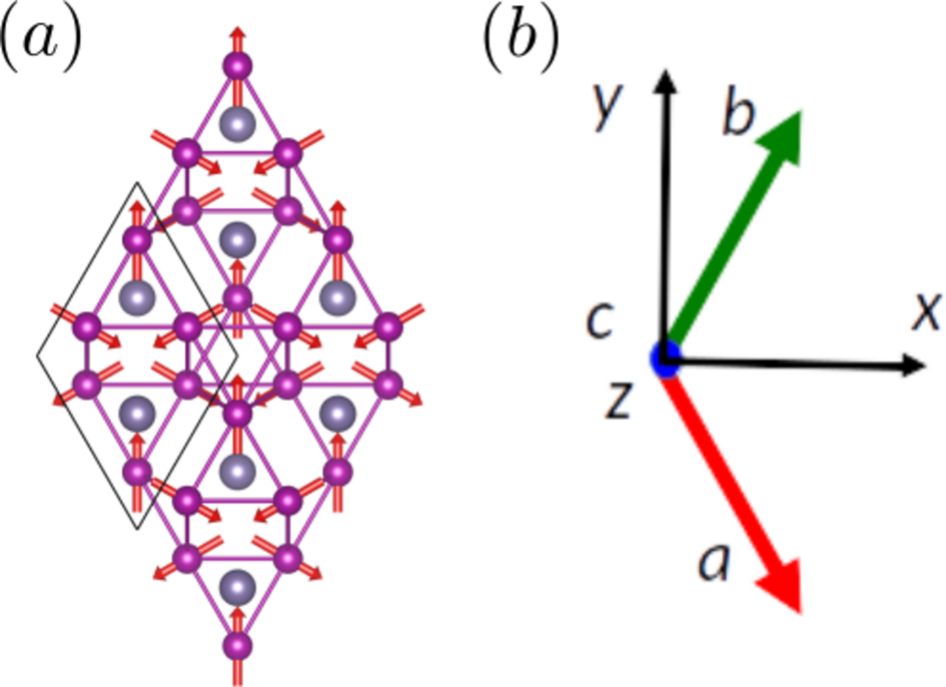
(*a*) Magnetic structure of Mn_3_Ge, showing the spins of the Mn atoms. (*b*) Relationship between the hexagonal unit-cell vectors 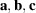 and the orthorhombic *xyz* directions of the basis unit vectors used to express the material tensors in the standard setting of its MSG, 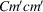.

The paramagnetic phase of Mn_3_Ge is hexagonal with space group 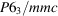 (No. 194). Below 380 K the material undergoes a transition to a coplanar magnetic structure (Soh *et al.*, 2020 ▸). The plane of spins is perpendicular to the hexagonal axis [see Fig. 4 ▸(*a*)], and the MSG of the structure is 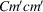 (OG No. 63.8.58). The corresponding MPG is 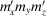, where the 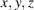 axes are associated with the orthorhombic unit cell 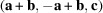 of the MSG standard unit cell. The relation of these orthorhombic axes with the crystallographic hexagonal 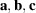 unit-cell vectors is depicted in Fig. 4 ▸(*b*).

The SpSG of this structure is a coplanar group with a non-trivial SpSG having the numerical index 11.194.1.2 (Chen *et al.*, 2024 ▸). The SpPG is generated by the following operations (not a minimal set, to facilitate comparison with the MPG): 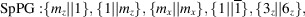
while the generators of the orthorhombic MPG are 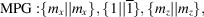
where we have used the same reference system of ortho­rhombic axes 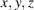 for both the spin and the space operations. The SpPG contains the MPG, as it should, and adds two additional generators: the threefold/sixfold rotation and the spin-only mirror plane. The requirement of tensor invariance for these two operations is sufficient to derive the additional constraints on the tensors under the SpPG. The MPG_eff_ corresponding to the above SpPG, to be considered for orbital contributions, is 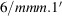. As in all coplanar and collinear structures, it is a gray magnetic group, which forbids any orbital contribution to any time-odd tensor.

Table 8 ▸ gathers a few examples of tensors, showing the difference in their symmetry-adapted forms under SpPG and MPG symmetries. Some comments on the results are in order. The SpPG does not allow the existence of spontaneous magnetization, unlike the MPG. This implies that the allowed ferromagnetism of this material, which is observed macroscopically as a weak feature (Soh *et al.*, 2020 ▸), has the SOC as the ultimate cause. Remarkably, the anomalous Hall effect, described by the antisymmetric terms of the resistivity, 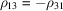, has been reported to be ‘giant’ (Kiyohara *et al.*, 2016 ▸), though it should also be a SOC effect, since it is allowed by the MPG and forbidden by the SpPG.

The electric and magnetic susceptibilities change from being diagonal in the MPG with three independent terms to having two of them equal in the SpPG, keeping the axial symmetry of the parent phase. A similar case happens with the ordinary Seebeck effect and the symmetric part of the electric resistivity, with a single additional constraint, 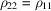, in the SpPG. The spin Hall resistivity 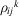 (antisymmetric part in the *ij* indices) also reduces from having three to only one independent coefficient. Note that the spin-only operation 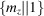, due to the coplanarity of the structure, is already sufficient to make the antisymmetric part of the spin resistivity vanish for *x* and *y* polarizations, 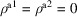 (see Table 5 ▸). On the other hand, the symmetric part of 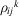 is also drastically reduced (five independent coefficients under the MPG versus one coefficient under the SpPG). In particular, 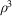 goes from being allowed in the MPG to being null in the SpPG, which can be attributed exclusively to the coplanar spin-only symmetry present in the SpPG.

#### 5.2.2. Non-coplanar DyVO_3_ (entry 0.106 in MAGNDATA)

**Figure 5:**
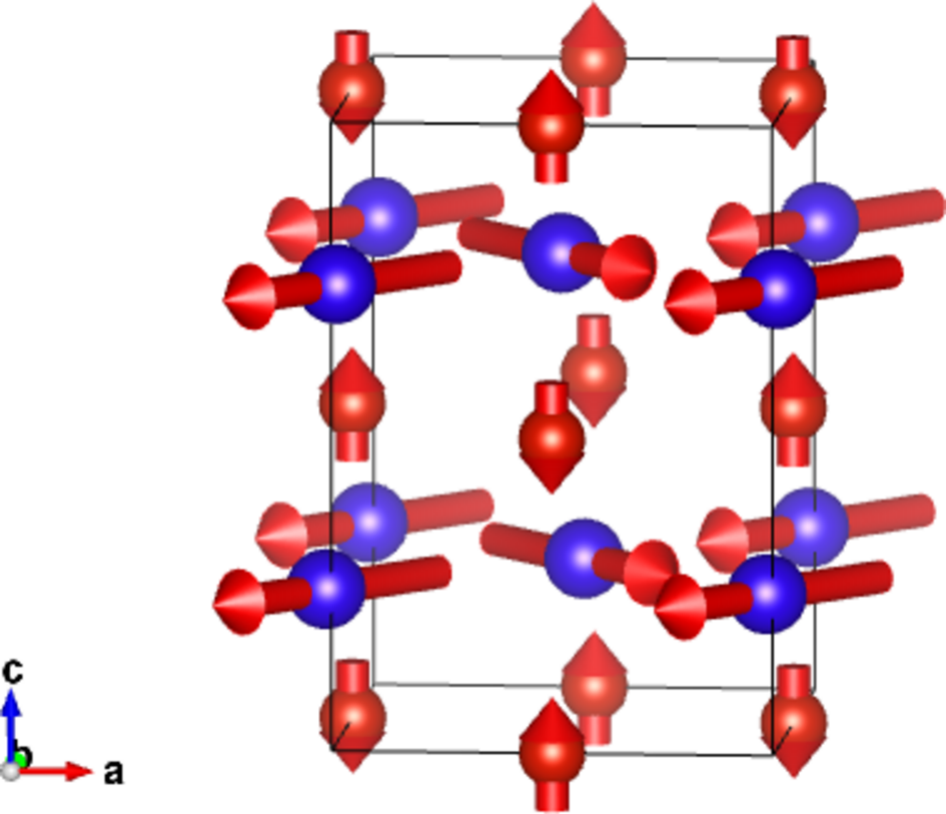
Magnetic structure of DyVO_3_ at 6 K showing only the magnetic atoms. Blue and red spheres represent Dy and V atoms, respectively.

This material has space group *Pbnm* (No. 62) in its paramagnetic phase. At low temperatures, both V and Dy atoms are magnetically ordered with a non-coplanar spin arrangement, which is depicted in Fig. 5 ▸ (Reehuis *et al.*, 2011 ▸). The MSG of this magnetic structure is 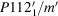 (OG No. 11.5.63). Being non-coplanar, the SpSG coincides with its non-trivial subgroup, which is denoted by the numerical label 2.62.1.8 by Chen *et al.* (2024 ▸). Thus, the SpSG, in contrast with the MSG, keeps all the space operations of the parent space group *Pbnm*, keeping an orthorhombic symmetry, while the MSG is monoclinic. Taking as reference system the *abc* crystallographic axes shown in Fig. 5 ▸, for both the spin and space operations, the corresponding SpPG can be denoted as 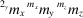, which can be identified with the non-trivial SpPG with number 81 in the listing of Litvin (1977 ▸), if the labeling of the axes in the spin space is changed. As generators of this SpPG we can take
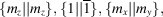
whereas the MPG (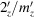) is generated by the first two of these three generators. Thus, the MPG is a subgroup of the SpPG, which is obtained from the former by just adding an additional generator.

With regard to the effective symmetry for the orbital contributions within the SpSG formalism, it is straightforward to derive using the SpPG generators listed above that MPG_eff_ = 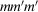.

We can now review a series of tensor properties and compare their symmetry-adapted forms according to both the MPG and SpPG.

A first simple example is the spontaneous magnetization. It readily follows that the MPG allows a magnetization of the form 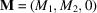. If considered under the SpPG, as only the *U* operations are involved in the transformations for the spin contribution to the magnetization, it can easily be seen by just inspecting the above-mentioned generators that under the SpPG the spin magnetization is restricted to the *y* direction, *i.e.*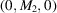. The magnitude of the magnetization along this direction is in fact very important, as can be seen in Fig. 5 ▸. In contrast, any additional spin magnetization along *x*, which is also allowed by the MPG, if present, would necessarily be a SOC effect and would break the SpPG assigned to the structure. Note, however, that a non-zero magnetization 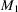 is allowed to exist without SOC, and under the same SpPG, but with the condition that it must be of orbital origin. Indeed, as MPG_eff_ = 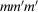, the orbital contribution to the magnetization must be of the form 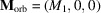, which should be added to the spin magnetization allowed along *y*.

This is an example of the problem, which was mentioned in Section 2, that may arise in practice, when the SpSG of an experimentally determined magnetic structure is identified. Let us consider the hypothetical case of a structure like the one in this example, with negligible SOC, but with a significant orbital contribution to the atomic moments of orbital origin, resulting in a non-zero magnetization of orbital origin along *x*, as permitted by the SpSG. As the SpSG symmetry is usually determined assuming that atomic magnetic moments have only spin contributions, the observed magnetic ordering would be considered incompatible with the actual SpSG of the structure, and instead a wrong SpSG will be assigned.

Another only-*U* tensor is the spin contribution to the magnetic susceptibility 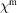 [see equation (21) and Table 2 ▸]. As the SpPG maintains the orthorhombic symmetry, it is constrained to be of the form 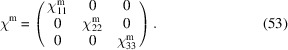
Considering the corresponding MPG_eff_, it is clear that the orbital contribution must have a similar diagonal form. Note, however, that, according to the rigorous definition of the SpPG, the diagonal directions *x*, *y* and *z* of the tensor in equation (53) refer only to the spin arrangement, while the diagonal axes of the orbital magnetic susceptibility are the crystallographic ones. In the SpSG formalism, the spin arrangement is considered unlocked from the lattice, and its global orientation is assumed to be arbitrary. Hence, if the SpPG concept is taken literally, the two diagonal tensors of spin and the orbital magnetic susceptibilities refer in general to two different systems of axes. But the clear locking between lattice and spins in a real case as this, obvious in Fig. 5 ▸, makes it necessary that the reference axes for the spins are chosen coincident with the crystallographic ones, as we did in the description of the SpPG.

As the MPG is monoclinic, the magnetic susceptibility under this lower symmetry also includes non-diagonal terms, namely the coefficient 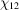, since the monoclinic axis is along *z*. Thus, the tensor deviation from the orthorhombic prescribed diagonal form, also valid for the paramagnetic phase, is expected to be a SOC effect.

The same reduction as in equation (53) happens with other second-rank tensors like, for example, the static electric susceptibility 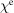. Although the Jahn symbol of this tensor is the same for the MPG as for the SpPG ([V^2^]), the final form of the reduced tensor is different because of the presence of the extra space operation in the SpPG. Identical conclusions are reached for the symmetric part of the electric resistivity tensor ρ or the symmetric part of the optical dielectric tensor ɛ, since their Jahn symbols are [V^2^] in all cases (see Tables S3 and S5 in the supporting information).

The antisymmetric parts of the electric resistivity tensor ρ and optical dielectric tensor ɛ also have different forms for the MPG and SpPG (see the last columns in Tables S3 and S5 in the supporting information). The final symmetry-adapted forms of the tensors are 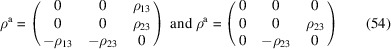
for the MPG and SpPG, respectively, and equivalent forms for the antisymmetric part of the optical dielectric tensor. The second of equations (54) shows that even under the SpPG symmetry, and therefore with negligible SOC, the anomalous Hall effect (and the spontaneous Faraday effect) are permitted in the material. The 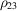 component is the so-called geometric part of the anomalous Hall effect, whereas 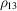 is a Karplus–Luttinger term, as it is SOC-assisted (Watanabe *et al.*, 2024 ▸).

Further tensor properties of this material are presented in the supporting information (Section S4) along with other example materials: non-coplanar CaFe_3_Ti_4_O_12_ and collinear UCr_2_Si_2_C (Sections S5 and S6, respectively).

## 6. Related literature

The following references are cited in the supporting information: Kleinman (1962 ▸), Klyshko (2011 ▸), Lemoine *et al.* (2018 ▸), Patino *et al.* (2021 ▸), Pershan (1963 ▸), Popov *et al.* (1995 ▸), Tsirkin & Souza (2022 ▸).

## 7. Conclusions

In this paper we present a general formalism for the derivation of the symmetry-adapted form of any crystal tensor property of a magnetic material considering its SpPG. We have stressed the important fact that a null SOC is required for a SpSG to be rigorously considered as a symmetry group of a magnetic structure. This means that SpSGs should be considered in most real cases as approximate symmetries. In order to compare tensor constraints under SpSG symmetry with those under the actual magnetic group of the structure, both the spin and magnetic groups must be described within a common framework, where they have a group–subgroup relation. This implies the choice of a specific orientation of the spin arrangement with respect to the lattice, consistent with the observed structure. In this way, SOC-free tensor properties, permitted by the SpPG symmetry, can be systematically distinguished from those having necessarily SOC as their ultimate cause.

After reviewing the mathematical structure of SpSGs and SpPGs and their relation with ordinary MSGs and MPGs, the symmetry conditions to be satisfied by crystalline tensors under a SpPG have been analyzed. More specifically, we have carried out a systematic study of the specific action that a 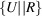 operation of a SpPG produces on various types of tensors describing macroscopic physical properties of magnetic structures. The transformation laws obtained constitute a generalization of the laws corresponding to the MPG operations, which are particular cases when 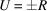. Using a generalization of the Neumann principle to SpPGs we have found the restrictions that the SpPG symmetry imposes on four types of tensors, describing equilibrium, transport, optical and second-order NLO properties. To each tensor property we have assigned a symbol, which generalizes the Jahn symbols for the MPGs and summarizes its transformation properties under a general operation 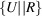.

We demonstrate that the spin-only symmetry, which is intrinsic in the SpPG of all collinear or coplanar magnetic structures, introduces very general constraints on the tensors when SOC-free SpPG symmetry is assumed. It is worth noting that, in most practical cases (about 75% of the reported structures), the SpSG only adds the spin-only symmetry and, therefore, the general collinear-based or coplanar-based constraints are the only extra restrictions to be added to the constraints resulting from the MPG. Finally, we illustrate the effects of the SpPG symmetries on various tensor properties for more complex SpPG–MPG relations by analyzing several examples of representative materials with non-coplanar, coplanar and collinear magnetic orderings.

A word of caution is in order regarding the way that the formalism presented in this work can be applied to an experimentally determined magnetic structure. The identification of the MSG of a given structure is a well defined mathematical process, with no additional assumption needed, except that the structure is correct. But the determination of its SpSG, as its alternative symmetry group in the case that the SOC is null, has some ambiguities. The SpSGs of practically all commensurate magnetic structures available in the MAGNDATA database have been calculated and reported in several works (Chen *et al.*, 2024 ▸; Jiang *et al.*, 2024 ▸; Xiao *et al.*, 2024 ▸). However, these SpSG identifications were done with the implicit assumption that the spin arrangement does not have any feature caused by the SOC that would falsify the calculated SpSG. This is usually quite a reasonable assumption because, except for the magnetic anisotropy that locks the global orientation of the spin arrangement with respect to the lattice, structural effects with SOC origin are usually weak. In many cases, they are not detectable by the typical neutron diffraction techniques employed in magnetic structure determination. However, this assumption sometimes fails, for instance, when the structure includes some small but significant spin canting of SOC origin. As an example, if one inspects Fig. 3 ▸, one may suspect that the deviation of the structure from collinearity is a local locking effect, which requires a non-zero SOC. Thus, there are experimental structures whose assigned SpSG is a subgroup of the resulting SpSG if the SOC contribution were not considered (no canting in the example above), and the distinction between SOC-free and SOC-based tensor properties using the assigned SpSG would be wrong. The SpSGs identified from MAGNDATA entries also assumed that the magnetic orderings have no orbital contribution or are irrelevant for the SpSG determination. We have seen above that in collinear structures the associated SpSG symmetry forbids in any case any orbital contribution to the atomic spins. There are, however, collinear structures with a demonstrated significant contribution to the atomic moments due to SOC effects. Hence, in such cases, ignoring the presence of the orbital contribution paradoxically allows one to assign the correct SOC-free SpSG.

Regardless of whether the calculated SpSG of an experimentally determined magnetic structure is or is not the SOC-free symmetry group of the system, it might be tempting to consider this group as a ‘geometric’ symmetry feature, which could be applied to derive the symmetry constraints for any property of the material. This would be, however, wrong. If the tensor constraints dictated by the identified SpSG symmetry were taken as exact, then many important observations would remain unexplained, such as the weak ferromagnetism in collinear or coplanar structures, the magnetically induced electric polarization found in many multiferroics, or the significant orbital contribution present in some collinear structures. In summary, SpSG symmetry should not be generally taken as the real symmetry of a structure, but as a good approximation, which allows one to separate, as shown in this work, those features and properties in the system which are not caused by the SOC, and therefore are especially important.

To end this paper, we would like to announce that we have recently developed a computer program (*STENSOR*) that, following the approach presented in this article, permits an automatic calculation of symmetry-adapted tensors under a given oriented SpPG and its comparison with their form under the corresponding MPG. It is open access and has been incorporated in the Bilbao Crystallographic Server (https://cryst.ehu.es/cryst/stensor.html).

## Supplementary Material

Further tables and examples. DOI: 10.1107/S2053273325004127/cam5007sup1.pdf

## Figures and Tables

**Table 1:** Transformation of the four basic ferroic effects under the space-inversion and time-reversal operations The four effects are denoted by the symbols V, *e*V, M and T. Effects V and T are odd for the space inversion, while *e*V and M are even. For the time reversal V and *e*V are even, while M and T are odd.

	V	*e*V	M	T
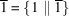	−1	1	1	−1
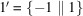	1	1	−1	−1

**Table 2:** Selection of some equilibrium properties with their Jahn symbols for the MPGs and SpPGs and their transformation laws under the SpPG Only tensors related to spin magnetism are listed (see text). 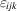 is the Levi-Civita symbol, and 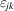 and 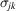 stand for the strain and stress tensors, respectively. In the case of MPGs the label *e* in the Jahn symbol indicates an axial tensor and the label *a* a magnetic tensor, *i.e.* odd for time reversal. This means that the law of tensor transformation includes a change of sign for improper operations (*e*) or for operations that include time reversal (*a*). The square brackets and curly brackets indicate symmetry and antisymmetry of pairs of indices, respectively. The symmetric or antisymmetric character is not explicit in the outline of the transformation law indicated in the last column.

Tensor description	Defining equation	Jahn symbol (MPG/SpPG)	Transformation laws (SpPG)
Magnetization	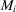	*ae*V/M	*UM*
Polar toroidic moment	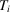	*a*V/{MV}	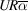 ; 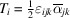
Magnetic susceptibility tensor 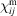	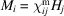	[V^2^]/[M^2^]	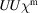
Magnetoelectric tensor 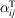 (inverse effect)	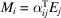	*ae*V^2^/MV	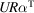
Electrotoroidic tensor 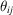 (inverse effect)	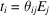	*a*V^2^/{MV}V	*URRb*; 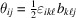
Piezotoroidic tensor 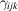 (direct effect)	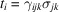	*a*V[V^2^]/{MV}[V^2^]	*URRRb*; 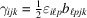
Second-order magnetoelectric tensor 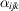 (direct effect)	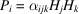	V[V^2^]/V[M^2^]	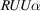
Piezomagnetic tensor 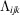 (direct effect)	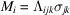	*ae*V[V^2^]/ M[V^2^]	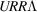
Magnetostriction tensor 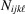	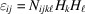	[V^2^][V^2^]/[V^2^][M^2^]	*RRUUN*

**Table 3:** Constraints imposed by collinearity and coplanarity on some magnetic tensors of equilibrium properties, assuming SpPG symmetry Only tensors related to spin magnetism are listed. The *z* direction is taken as the spin direction in the collinear case and as the direction perpendicular to the spin planes in the coplanar case.

Tensor	Collinear structure	Coplanar structure
Magnetization 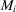 (spin contribution)	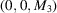	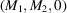
Toroidic moment 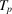 (spin contribution) 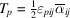	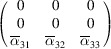	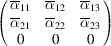
Magnetic susceptibility 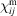 (spin contribution)	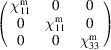	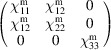
Magnetoelectric tensor 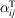 (spin contribution) (inverse effect)	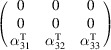	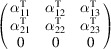
Electrotoroidic tensor 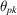 (spin contribution) (inverse effect) 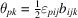	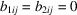 , 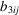 no restriction	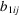 , 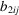 no restriction, 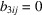
Piezotoroidic tensor 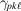 (spin contribution) (direct effect) 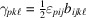	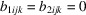 , 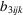 no restriction	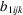 , 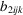 no restriction, 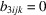
Second-order magnetoelectric tensor 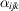 (spin contribution) (direct effect)	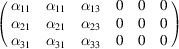	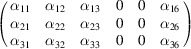
Piezomagnetic tensor 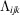 (spin contribution) (direct effect)	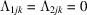 , 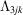 no restriction	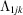 , 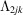 no restriction, 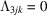
Magnetostriction tensor 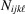 ( 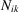 in abbreviated notation) (spin contribution)	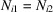 , 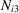 arbitrary, 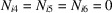 ; 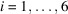	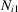 , 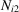 , 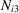 , 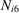 arbitrary, 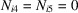 ; 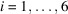

**Table 4:** Selected examples of the spin contributions of some transport tensors and their Jahn symbols in the context of MPGs and SpPGs For the SpPGs the transformation laws that each Jahn symbol implies are also given.

Tensor description	Defining equation	Jahn symbol (MPG/SpPG)	Transformation laws (SpPG)
Hall effect tensor 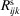 (symmetric part) Linear magnetoresistance	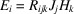 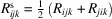	*ae*[V^2^]V/[V^2^]M	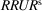 (Spin)
Hall effect tensor 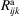 (antisymmetric part) Ordinary Hall effect	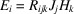 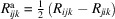	*e*{V^2^}V/*a*{V^2^}M	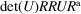 (Spin)
Spin/orbital Hall resistivity tensor 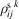 (symmetric part)	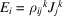 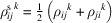	*ae*[V^2^]V/[V^2^]M	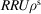 (Spin)
Spin/orbital Hall resistivity tensor 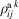 (antisymmetric part)	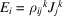 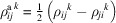	*e*{V^2^}V/*a*{V^2^}M	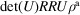 (Spin)

**Table 5:** Constraints imposed by collinearity and coplanarity on the spin contributions of some tensors for transport phenomena assuming SpPG symmetry The *z* direction is chosen as in Table 2 ▸ to define the orientation of the spins or the spin planes.

Tensor	Collinear structure	Coplanar structure
Hall effect tensor 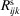 (symmetric part) (spin contribution) Linear magnetoresistance	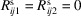 , 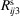 no restriction	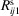 , 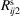 no restriction, 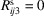
Hall effect tensor 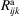 (antisymmetric part) (spin contribution) Ordinary Hall effect	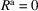	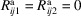 , 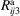 no restriction
Spin Hall resistivity tensor 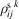 (symmetric part)	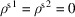 , 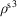 no restriction	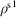 , 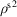 no restriction, 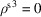
Spin Hall resistivity tensor 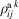 (antisymmetric part)	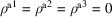	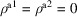 , 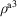 no restriction

**Table 6:** Spin contribution to the Faraday effect tensors with their Jahn symbols in the context of MPGs and SpPGs, and their transformation laws under an SpPG operation

Tensor description	Defining equation	Jahn symbol (MPG/SpPG)	Transformation laws (SpPG)
Faraday effect tensor 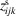 (symmetric part) Magneto-optic Kerr effect (MOKE)	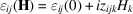 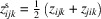	*ae*[V^2^]V/[V^2^]M	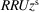 (Spin)
Faraday effect tensor 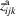 (antisymmetric part) Ordinary Faraday effect	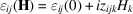 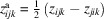	*e*{V^2^}V/*a*{V^2^}M	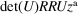 (Spin)

**Table 7:** Constraints imposed by collinearity and coplanarity on the spin contributions to the Faraday tensors assuming SpPG symmetry

Tensor	Collinear structure	Coplanar structure
Faraday effect tensor 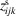 (symmetric part) (spin contribution) Magneto-optic Kerr effect (MOKE)	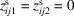 , 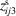 no restriction	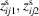 no restriction, 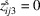
Faraday effect tensor 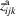 (antisymmetric part) (spin contribution) Ordinary Faraday effect	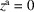	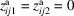 , 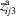 no restriction

**Table 8:** Comparison of symmetry-adapted tensor forms of some selected tensor properties in the magnetic phase of Mn_3_Ge according to the magnetic and spin point groups

Tensor property	MPG	SpPG
Magnetization	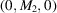	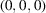
Magnetic susceptibility/electric susceptibility	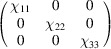	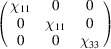
		
Electric resistivity	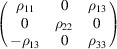	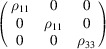
		
Spin Hall resistivity (symmetric part)	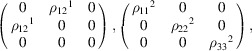	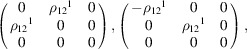
	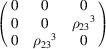	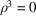
		
Spin Hall resistivity (antisymmetric part)	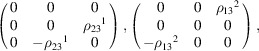	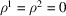
	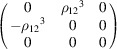	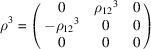
		
Ordinary Seebeck effect 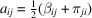	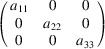	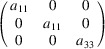
		
Spontaneous Nernst effect 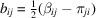	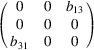	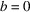

## Appendix A Glossary of some important groups used in the article and their notation

G_SS_ Full spin space group (SpSG) formed by operations .

G_SO_ Spin-only space group. Subgroup of G_SS_ formed by operations .

G_NT_ Non-trivial SpSG. Subgroup of G_SS_ formed by operations  with  for those operations with . For collinear structures  (Shubnikov-like group) by convention. For coplanar structures , proper operation, by convention.

Collinear G_SO_ with spin direction along **n**.

Coplanar G_SO_ with spin plane normal along **n**.

L_0_ Space group formed by operations  such that  G_NT_.

G_0_ Space group formed by all operations  such that G_NT_. G_0_ and L_0_ are related by G_0_ = L_0_ +  L_0_ + … +  L_0_, with  coset representatives.

G_ST_ Spin-translation group of G_NT_. Subgroup of G_NT_ formed by operations  with  not being a lattice translation if .

G_P_ Space group of the paramagnetic phase.

MSG Magnetic space group. Subgroup of G_SS_ with operations .

F Family space group of a MSG. Space group formed by all operations  present in the MSG, irrespective of whether or not they include time reversal.

P_S_ Spin point group (SpPG) formed by operations .

P_SO_ Spin-only point group. Subgroup of P_S_ formed by operations .

P_SOintr_ Intrinsic spin-only point group, P_SOintr_ =  for collinear and P_SOintr_ =  for coplanar groups.

P_SOG_ Subgroup of P_SO_. Intersection between P_SO_ and the set of operations  such that G_ST_. It is a proper subgroup of P_SO_ if the spin translation group G_ST_ contains elements with . Relation between P_SO_ and P_SOG_, P_SO_ = P_SOintr_ × P_SOG_.

P_NT_ Non-trivial spin point group. Subgroup of P_S_ formed by operations  with  except for the identity.

P_M_ Magnetic point group (MPG). Subgroup of P_S_ with operations . For minimal SpSGs, *i.e.* when SpSG and MSG have the same operations , the relation between P_M_ and P_S_ is P_S_ = P_M_ × P_SOintr_.

MPG_eff_ Effective magnetic point group of a SpPG with elements . It is the magnetic point group formed by operations .

## References

[bb1] Aroyo, M. I. (2016). Editor. *International tables for crystallography*, Vol. *A*, *Space-group symmetry*. Chester: International Union of Crystallography.

[bb2] Bernevig, B. A., Hughes, T. L. & Zhang, S.-C. (2005). *Phys. Rev. Lett.* **95**, 066601. 10.1103/PhysRevLett.95.066601 16090968

[bb3] Bhowal, S. & Spaldin, N. A. (2024). *Phys. Rev. X* **14**, 011019.

[bb4] Birss, R. R. (1963). *Rep. Prog. Phys.* **26**, 307–360.

[bb5] Brinkman, W. F. & Elliott, R. J. (1966). *Proc. R. Soc. Lond. A* **294**, 343–358.

[bb6] Butzal, H.-D. & Birss, R. R. (1982). *Physica A* **114**, 518–521.

[bb7] Campbell, B. J., Stokes, H. T., Perez-Mato, J. M. & Rodríguez-Carvajal, J. (2022). *Acta Cryst.* A**78**, 99–106. 10.1107/S2053273321012912 35230265

[bb8] Campbell, B. J., Stokes, H. T., Perez-Mato, J. M. & Rodriguez-Carvajal, J. (2024). *Acta Cryst.* B**80**, 401–408. 10.1107/S2052520624008084 39320310

[bb9] Chen, X., Ren, J., Zhu, Y., Yu, Y., Zhang, A., Liu, P., Li, J., Liu, Y., Li, C. & Liu, Q. (2024). *Phys. Rev. X* **14**, 031038.

[bb10] Cracknell, A. P. (1973). *Phys. Rev. B* **7**, 2145–2154.

[bb11] Eremenko, V. V., Kharchenko, N. F., Litvinenko, Y. G. & Naumenko, V. M. (1992). *Magneto-optics and spectroscopy of antiferromagnets*. New York: Springer.

[bb12] Frazer, B. C. & Brown, P. J. (1962). *Phys. Rev.* **125**, 1283–1291.

[bb13] Gallego, S. V., Etxebarria, J., Elcoro, L., Tasci, E. S. & Perez-Mato, J. M. (2019). *Acta Cryst.* A**75**, 438–447. 10.1107/S2053273319001748 31041900

[bb14] Gallego, S. V., Perez-Mato, J. M., Elcoro, L., Tasci, E. S., Hanson, R. M., Momma, K., Aroyo, M. I. & Madariaga, G. (2016). *J. Appl. Cryst.* **49**, 1750–1776.

[bb15] Grimmer, H. (1993). *Acta Cryst.* A**49**, 763–771.

[bb16] Grimmer, H. (1994). *Ferroelectrics* **161**, 181–189.

[bb17] Grimmer, H. (2017). *Acta Cryst.* A**73**, 333–345. 10.1107/S2053273317005368 28660865

[bb18] Hellenes, A. B., Jungwirth, T., Jaeschke-Ubiergo, R., Chakraborty, A., Sinova, J. & Šmejkal, L. (2024). arXiv:2309.01607. 10.1038/s41467-025-62516-0 PMC12332020 40775210

[bb19] Jahn, H. A. (1949). *Acta Cryst.* **2**, 30–33.

[bb20] Janssen, T., Janner, A., Looijenga-Vos, A. & de Wolf, P. M. (2004). *International tables for crystallography*, Vol. *C*, *Mathematical, physical and chemical tables*, edited by E. Prince, pp. 907–955. Dordrecht: Kluwer Academic Publishers.

[bb21] Jiang, Y., Song, Z., Zhu, T., Fang, Z., Weng, H., Liu, Z.-X., Yang, J. & Fang, C. (2024). *Phys. Rev. X* **14**, 031039.

[bb22] Kiyohara, N., Tomita, T. & Nakatsuji, S. (2016). *Phys. Rev. Appl.* **5**, 064009.

[bb23] Kleiner, W. H. (1966). *Phys. Rev.* **142**, 318–326.

[bb60] Kleinman, D. A. (1962). *Phys. Rev.* **126**, 1977–1979.

[bb61] Klyshko, D. N. (2011). *Physical foundations of quantum electronics*. Singapore: World Scientific.

[bb24] Kopský, V. (2015). *Symmetry (Basel)* **7**, 125–145.

[bb25] Landau, L. D. & Lifshitz, E. M. (1960). *Electrodynamics of continuous media.* Course of Theoretical Physics. London: Pergamon Press.

[bb62] Lemoine, P., Vernière, A., Pasturel, M., Venturini, G. & Malaman, B. (2018). *Inorg. Chem.* **57**, 2546–2557. 10.1021/acs.inorgchem.7b02901 29431434

[bb26] Litvin, D. B. (1977). *Acta Cryst.* A**33**, 279–287.

[bb27] Litvin, D. B. (2013). *Magnetic group tables: 1-2- and 3-dimensional magnetic subperiodic groups and space groups.* Chester: International Union of Crystallography.

[bb28] Litvin, D. B. (2016). *Magnetic subperiodic groups and magnetic space groups.* Chester: International Union of Crystallography.

[bb29] Litvin, D. B. & Opechowski, W. (1974). *Physica* **76**, 538–554.

[bb30] Liu, P., Li, J., Han, J., Wan, X. & Liu, Q. (2022). *Phys. Rev. X* **12**, 021016.

[bb31] Mazin, I. (2022). *Phys. Rev. X* **12**, 040002.

[bb32] Nagaosa, N., Sinova, J., Onoda, S., MacDonald, A. H. & Ong, N. P. (2010). *Rev. Mod. Phys.* **82**, 1539–1592.

[bb33] Nye, J. F. (1985). *Physical properties of crystals.* Oxford Science Publications. London: Oxford University Press.

[bb63] Patino, M. A., Romero, F. D., Goto, M., Saito, T., Orlandi, F., Manuel, P., Szabó, A., Kayser, P., Hong, K. H., Alharbi, K. N., Attfield, J. P. & Shimakawa, Y. (2021). *Phys. Rev. Res.* **3**, 043208.

[bb34] Pérez-Mato, J. M., Madariaga, G. & Tello, M. J. (1984). *Phys. Rev. B* **30**, 1534–1543.

[bb64] Pershan, P. S. (1963). *Phys. Rev.* **130**, 919–929.

[bb65] Popov, S., Svirko, Y. & Zheludev, N. (1995). *Susceptibility tensors for nonlinear optics*. Series in optics and optoelectronics. Taylor & Francis.

[bb35] Radaelli, P. G. (2024). *Phys. Rev. B* **110**, 214428.

[bb36] Reehuis, M., Ulrich, C., Prokeš, K., Mat’aš, S., Fujioka, J., Miyasaka, S., Tokura, Y. & Keimer, B. (2011). *Phys. Rev. B* **83**, 064404.

[bb37] Seemann, M., Ködderitzsch, D., Wimmer, S. & Ebert, H. (2015). *Phys. Rev. B* **92**, 155138.

[bb38] Shtrikman, S. & Thomas, H. (1965). *Solid State Commun.* **3**, 147–150.

[bb39] Šmejkal, L., Sinova, J. & Jungwirth, T. (2022*a*). *Phys. Rev. X* **12**, 031042.

[bb40] Šmejkal, L., Sinova, J. & Jungwirth, T. (2022*b*). *Phys. Rev. X* **12**, 040501.

[bb41] Soh, J.-R., de Juan, F., Qureshi, N., Jacobsen, H., Wang, H.-Y., Guo, Y.-F. & Boothroyd, A. T. (2020). *Phys. Rev. B* **101**, 140411.

[bb42] Spaldin, N. A., Fiebig, M. & Mostovoy, M. (2008). *J. Phys. Condens. Matter* **20**, 434203.

[bb43] Taguchi, Y., Oohara, Y., Yoshizawa, H., Nagaosa, N. & Tokura, Y. (2001). *Science* **291**, 2573–2576. 10.1126/science.1058161 11283363

[bb66] Tsirkin, S. S. & Souza, I. (2022). *SciPost Phys. Core* **5**, 039.

[bb44] Watanabe, H., Shinohara, K., Nomoto, T., Togo, A. & Arita, R. (2024). *Phys. Rev. B* **109**, 094438.

[bb45] Will, G. & Schafer, W. (1979). *J. Less-Common Met.* **67**, 31–39.

[bb46] Xiao, Z., Zhao, J., Li, Y., Shindou, R. & Song, Z.-D. (2024). *Phys. Rev. X* **14**, 031037.

[bb47] Yamani, Z., Tun, Z. & Ryan, D. H. (2010). *Can. J. Phys.* **88**, 771–797.

[bb48] Yuan, L.-D., Wang, Z., Luo, J.-W., Rashba, E. I. & Zunger, A. (2020). *Phys. Rev. B* **102**, 014422.

[bb49] Yuan, L.-D., Wang, Z., Luo, J.-W. & Zunger, A. (2021). *Phys. Rev. Mater.* **5**, 014409.

[bb50] Železný, J., Zhang, Y., Felser, C. & Yan, B. (2017). *Phys. Rev. Lett.* **119**, 187204. 10.1103/PhysRevLett.119.187204 29219584

[bb51] Zhang, Y., Železný, J., Sun, Y., van den Brink, J. & Yan, B. (2018). *New J. Phys.* **20**, 073028.

[bb52] Zhu, H., Li, J., Chen, X., Yu, Y. & Liu, Q. (2024). arXiv:2406.03738.

