# Supplementary material for: Crystal tensor properties of magnetic materials with and without spin–orbit coupling. Application of spin point groups as approximate symmetries
Source: Acta Crystallogr A Found Adv. 2025 Jun 10;81(Pt 4):317–38. doi: 10.1107/S2053273325004127 (PMC12207915; doi:10.1107/S2053273325004127)
Supplement: Supplementary file 1 [file a-81-00317-sup1.pdf]

FOUNDATIONS ADVANCES

Volume 81 (2025)

Supporting information for article:

Crystal tensor properties of magnetic materials with and without spin-orbit coupling. Application of spin point groups as approximate symmetries

Jesus Etxebarria, J. Manuel Perez-Mato, Emre S. Tasci and Luis Elcoro

Supporting information

Crystal tensor properties of magnetic materials with and without spin-orbit coupling.

Application of spin point groups as approximate symmetries.

Jesus Etxebarria[a], J. Manuel Perez-Mato[b], Emre S. Tasci[c], and Luis Elcoro[a]

[a]Department of Physics, Faculty of Science and Technology, UPV/EHU, Bilbao, Spain
[b]Faculty of Science and Technology, UPV/EHU, Bilbao, Spain
[c]Department of Physics Engineering, Hacettepe University, 06800 Ankara, Turkey

Contents

S1 Tables (S1-S6) of tensor properties and constraints due to spin group symmetry and glossary of groups used **3**

S1.1 Tensors of selected equilibrium properties . . . 3
S1.2 Constraints imposed by collinearity and coplanarity on equilibrium properties . . . 4
S1.3 Tensors of selected transport properties . . . 6
S1.4 Constraints imposed by collinearity and coplanarity on transport properties . . . 7
S1.5 Tensors of selected optical properties . . . 8
S1.6 Constraints imposed by collinearity and coplanarity on optical properties . . . 9

S2 Nonlinear optical properties **10**

S3 Constraints on tensors for nonlinear optical susceptibilities of collinear and coplanar magnetic structures **11**

S4 Study of further properties in non-coplanar $DyVO_3$ (entry 0.106 in MAGNDATA) **11**

S5 Non-coplanar $CaFe_3Ti_4O_{12}$ (entry 3.24 in MAGNDATA) **13**

S6 Collinear UCr_2Si_2C (entry 0.499 in MAGNDATA) **15**

S1 Tables (S1-S6) of tensor properties and constraints due to spin group symmetry and glossary of groups used

S1.1 Tensors of selected equilibrium properties

Table S1: Selection of some equilibrium properties with their Jahn symbols for the MPGs and SpPGs (added only when it is different from the symbol of the MPG), and their transformation laws under the SpPG. ε_{ij} and σ_{jk} stand for the strain and stress tensors respectively. The symbol ε in the last column stands for the Levi-Civita symbol and $\overline{\alpha}$ and b are the rank-2 and rank-3 tensors defined in sections 4.3 and 4.4 of the main text, respectively. In the case of MPGs, the label e in the Jahn symbol indicates an axial tensor and the label a a magnetic tensor, i.e., odd for time reversal. This means that the law of tensor transformation adds a change of sign for improper operations (e) or for operations that include time reversal (a). Where applicable, orbital and spin contributions have been separated.

**Tensor description**	**Defining Equation**	**Jahn Symbol (MPG/SpPG)**	**Transformation laws (SpPG)**
Polarization	P_i	V	RP
Magnetization	M_i	$a e$V/M	UM (Spin)<br>$\det(U)\det(R)RM$ (Orbital)
Polar Toroidic moment	T_i	aV/{MV}	$UR\overline{\alpha}$; $T=\frac{1}{2}\varepsilon\overline{\alpha}$ (Spin)<br>$\det(U)RT$ (Orbital)
Axial Toroidic moment	A_i	eV	$\det(R)RA$
Dielectric susceptibility tensor χ^e_{ij}	$P_i=\chi^e_{ij}E_j$	[V^2]	$RR\chi^e$
Magnetic susceptibility tensor χ^m_{ij}	$M_i=\chi^m_{ij}H_j$	[V^2]/[M^2]	$UU\chi^m$ (Spin)<br>$RR\chi^m$ (Orbital)
Magnetoelectric tensor α^T_{ij} (inverse effect)	$M_i=\alpha^T_{ij}E_j$	$a e$V^2/MV	$UR\alpha^T$ (Spin)<br>$\det(U)\det(R)RR\alpha^T$ (Orbital)
Electrotoroidic tensor θ_{ij} (inverse effect)	$t_i=\theta_{ij}E_j$	aV^2/{MV}V	$URRb$; $\theta=\frac{1}{2}\varepsilon b$ (Spin)<br>$\det(U)RRb$ (Orbital)
Piezoelectric tensor d_{ijk} (direct effect)	$P_i=d_{ijk}\sigma_{jk}$	V[V^2]	$RRRd$
Piezotoroidic tensor γ_{ijk} (direct effect)	$t_i=\gamma_{ijk}\sigma_{jk}$	aV[V^2]/{MV}[V^2]	$URRRb$; $\gamma=\frac{1}{2}\varepsilon b$ (Spin)<br>$\det(U)RRR\gamma$ (Orbital)
Second order magnetoelectric tensor α_{ijk} (direct effect)	$P_i=\alpha_{ijk}H_jH_k$	V[V^2]/V[M^2]	$RUU\alpha$ (Spin)<br>$RRR\alpha$ (Orbital)
Piezomagnetic tensor Λ_{ijk} (direct effect)	$M_i=\Lambda_{ijk}\sigma_{jk}$	$a e$V[V^2]/M[V^2]	$URR\Lambda$ (Spin)<br>$\det(U)\det(R)RRR\Lambda$ (Orbital)
Magnetostriction tensor $N_{ijk\ell}$	$\varepsilon_{ij}=N_{ijk\ell}H_kH_\ell$	[V^2][V^2]/[V^2][M^2]	$RRUUN$ (Spin)<br>$RRRRN$ (Orbital)

S1.2 Constraints imposed by collinearity and coplanarity on equilibrium properties

Table S2: Constraints imposed by collinearity and coplanarity on some tensors of equilibrium properties. Spin and orbital contributions have been separated when applicable.

**Tensor**	**Collinear structure**	**Coplanar structure**
Polarization P_i	no restriction	no restriction
Magnetization M_i<br>(spin contribution)	$(0, 0, M_3)$	$(M_1, M_2, 0)$
Magnetization M_i<br>(orbital contribution)	$\mathbf{M} = 0$	$\mathbf{M} = 0$
Toroidic moment T_p<br>(spin contribution)<br>$T_p = \frac{1}{2}\varepsilon_{pij}\overline{\alpha}_{ij}$	$\begin{pmatrix} 0 & 0 & 0 \\ 0 & 0 & 0 \\ \overline{\alpha}_{31} & \overline{\alpha}_{32} & \overline{\alpha}_{33} \end{pmatrix}$	$\begin{pmatrix} \overline{\alpha}_{11} & \overline{\alpha}_{12} & \overline{\alpha}_{13} \\ \overline{\alpha}_{21} & \overline{\alpha}_{22} & \overline{\alpha}_{23} \\ 0 & 0 & 0 \end{pmatrix}$
Toroidic moment T_i<br>(orbital contribution)	$\mathbf{T} = 0$	$\mathbf{T} = 0$
Axial Toroidic moment A_i	no restriction	no restriction
Dielectric susceptibility<br>tensor χ^e_{ij}	no restriction	no restriction
Magnetic susceptibility χ^m_{ij}<br>(spin contribution)	$\begin{pmatrix} \chi^m_{11} & 0 & 0 \\ 0 & \chi^m_{11} & 0 \\ 0 & 0 & \chi^m_{33} \end{pmatrix}$	$\begin{pmatrix} \chi^m_{11} & \chi^m_{12} & 0 \\ \chi^m_{12} & \chi^m_{22} & 0 \\ 0 & 0 & \chi^m_{33} \end{pmatrix}$
Magnetic susceptibility χ^m_{ij}<br>(orbital contribution)	no restriction	no restriction
Magnetoelectric tensor α^T_{ij}<br>(spin contribution)<br>(inverse effect)	$\begin{pmatrix} 0 & 0 & 0 \\ 0 & 0 & 0 \\ \alpha^T_{31} & \alpha^T_{32} & \alpha^T_{33} \end{pmatrix}$	$\begin{pmatrix} \alpha^T_{11} & \alpha^T_{12} & \alpha^T_{13} \\ \alpha^T_{21} & \alpha^T_{22} & \alpha^T_{23} \\ 0 & 0 & 0 \end{pmatrix}$
Magnetoelectric tensor α^T_{ij}<br>(orbital contribution)	$\alpha^T = 0$	$\alpha^T = 0$
Electrotoroidic tensor θ_{pk}<br>(spin contribution) (inverse effect)<br>$\theta_{pk} = \frac{1}{2}\varepsilon_{pij}b_{ijk}$	$b_{1ij} = b_{2ij} = 0$,<br>b_{3ij} no restriction	b_{1ij}, b_{2ij} no restriction,<br>$b_{3ij} = 0$
Electrotoroidic tensor θ_{ij}<br>(orbital contribution)	$\theta = 0$	$\theta = 0$
Piezoelectric tensor d_{ijk}	no restriction	no restriction
Piezotoroidic tensor $\gamma_{pk\ell}$<br>(spin contribution) (direct effect)<br>$\gamma_{pk\ell} = \frac{1}{2}\varepsilon_{pij}b_{ijk\ell}$	$b_{1ijk} = b_{2ijk} = 0$,<br>b_{3ijk} no restriction	b_{1ijk}, b_{2ijk} no restriction,<br>$b_{3ijk} = 0$

Table S2: Continued

**Tensor**	**Collinear structure**	**Coplanar structure**
Piezotoroidic tensor γ_{ijk} (orbital contribution)	$\gamma = 0$	$\gamma = 0$
Second order magnetoelectric tensor α_{ijk} (spin contribution) (direct effect)	$\begin{pmatrix} \alpha_{11} & \alpha_{11} & \alpha_{13} & 0 & 0 & 0 \\ \alpha_{21} & \alpha_{21} & \alpha_{23} & 0 & 0 & 0 \\ \alpha_{31} & \alpha_{31} & \alpha_{33} & 0 & 0 & 0 \end{pmatrix}$	$\begin{pmatrix} \alpha_{11} & \alpha_{12} & \alpha_{13} & 0 & 0 & \alpha_{16} \\ \alpha_{21} & \alpha_{22} & \alpha_{23} & 0 & 0 & \alpha_{26} \\ \alpha_{31} & \alpha_{32} & \alpha_{33} & 0 & 0 & \alpha_{36} \end{pmatrix}$
Second order magnetoelectric tensor α_{ijk} (orbital contribution)	no restriction	no restriction
Piezomagnetic tensor Λ_{ijk} (spin contribution) (direct effect)	$\Lambda_{1jk} = \Lambda_{2jk} = 0$, Λ_{3jk} no restriction	Λ_{1jk}, Λ_{2jk} no restriction, $\Lambda_{3jk} = 0$
Piezomagnetic tensor Λ_{ijk} (orbital contribution)	$\Lambda = 0$	$\Lambda = 0$
Magnetostriction tensor $N_{ijk\ell}$ (spin contribution)	$\begin{pmatrix} N_{11} & N_{11} & N_{13} & 0 & 0 & 0 \\ N_{21} & N_{21} & N_{23} & 0 & 0 & 0 \\ N_{31} & N_{31} & N_{33} & 0 & 0 & 0 \\ N_{41} & N_{41} & N_{43} & 0 & 0 & 0 \\ N_{51} & N_{51} & N_{53} & 0 & 0 & 0 \\ N_{61} & N_{61} & N_{63} & 0 & 0 & 0 \end{pmatrix}$	$\begin{pmatrix} N_{11} & N_{12} & N_{13} & 0 & 0 & N_{16} \\ N_{21} & N_{22} & N_{23} & 0 & 0 & N_{26} \\ N_{31} & N_{32} & N_{33} & 0 & 0 & N_{36} \\ N_{41} & N_{42} & N_{43} & 0 & 0 & N_{46} \\ N_{51} & N_{52} & N_{53} & 0 & 0 & N_{56} \\ N_{61} & N_{62} & N_{63} & 0 & 0 & N_{66} \end{pmatrix}$
Magnetostriction tensor $N_{ijk\ell}$ (orbital contribution)	no restriction	no restriction

S1.3 Tensors of selected transport properties

Table S3: Selected examples of transport tensors and their Jahn symbols in the context of MPGs and SpPGs. For the SpPGs, the transformation law that each Jahn symbol implies are also given. Some tensors have separated contributions coming from spin and orbital degrees of freedom. Seebeck and Peltier tensors β and π which appear in the last two rows are defined through equations $E_i = \beta_{ij} \nabla_j T$ and $q_i = \pi_{ij} E_j$. Ordinary Seebeck and Peltier tensors are transpose of each other, and the same relationship exists between spontaneous Nernst and spontaneous Ettingshausen tensors.

**Tensor description**	**Defining Equation**	**Jahn Symbol (MPG/SpPG)**	**Transformation laws (SpPG)**
Resistivity tensor ρ^s_{ij} (symmetric part) Ordinary resistivity	$E_i = \rho_{ij} J_j$<br>$\rho^s_{ij} = \frac{1}{2}(\rho_{ij} + \rho_{ji})$	$[\mathrm{V}^2]$	$RR\rho^s$
Resistivity tensor ρ^a_{ij} (antisymmetric part) Spontaneous Hall effect	$E_i = \rho_{ij} J_j$<br>$\rho^a_{ij} = \frac{1}{2}(\rho_{ij} - \rho_{ji})$	$a\{\mathrm{V}^2\}$	$\det(U)RR\rho^a$
Hall effect tensor R^s_{ijk} (symmetric part) Linear magnetoresistance	$E_i = R_{ijk} J_j H_k$<br>$R^s_{ijk} = \frac{1}{2}(R_{ijk} + R_{jik})$	$ae[\mathrm{V}^2]\mathrm{V}/[\mathrm{V}^2]\mathrm{M}$	$RRUR^s$ (Spin)<br>$\det(U)\det(R)RRRR^s$ (Orbital)
Hall effect tensor R^a_{ijk} (antisymmetric part) Ordirary Hall effect	$E_i = R_{ijk} J_j H_k$<br>$R^a_{ijk} = \frac{1}{2}(R_{ijk} - R_{jik})$	$e\{\mathrm{V}^2\}\mathrm{V}/a\{\mathrm{V}^2\}\mathrm{M}$	$\det(U)RRUR^a$ (Spin)<br>$\det(R)RRRR^a$ (Orbital)
Spin/orbital Hall resistivity tensor ${\rho^s_{ij}}^k$ (symmetric part)	$E_i = {\rho_{ij}}^k {J_j}^k$<br>${\rho^s_{ij}}^k = \frac{1}{2}\left({\rho_{ij}}^k + {\rho_{ji}}^k\right)$	$ae[\mathrm{V}^2]\mathrm{V}/[\mathrm{V}^2]\mathrm{M}$	$RRU\rho^s$ (Spin)<br>$\det(U)\det(R)RRR\rho^s$ (Orbital)
Spin/orbital Hall resistivity tensor ${\rho^a_{ij}}^k$ (antisymmetric part)	$E_i = {\rho_{ij}}^k {J_j}^k$<br>${\rho^a_{ij}}^k = \frac{1}{2}\left({\rho_{ij}}^k - {\rho_{ji}}^k\right)$	$e\{\mathrm{V}^2\}\mathrm{V}/a\{\mathrm{V}^2\}\mathrm{M}$	$\det(U)RRU\rho^a$ (Spin)<br>$\det(R)RRR\rho^a$ (Orbital)
Ordinary Seebeck effect<br>Ordinary Peltier effect	$\frac{1}{2}(\beta_{ij} + \pi_{ji})$<br>$\frac{1}{2}(\beta_{ji} + \pi_{ij})$	V^2	$RR\frac{1}{2}(\beta + \pi)$
Spontaneous Nernst effect<br>Spontaneous Ettingshausen effect	$\frac{1}{2}(\beta_{ij} - \pi_{ji})$<br>$\frac{1}{2}(\beta_{ji} - \pi_{ij})$	$a\mathrm{V}^2$	$\det(U)RR\frac{1}{2}(\beta - \pi)$

S1.4 Constraints imposed by collinearity and coplanarity on transport properties

Table S4: Constraints imposed by collinearity and coplanarity on some tensors for transport phenomena.

**Tensor**	**Collinear structure**	**Coplanar structure**
Resistivity tensor ρ^s_{ij}<br>(symmetric part)<br>Ordinary resistivity	no restrictions	no restrictions
Resistivity tensor ρ^a_{ij}<br>(antisymmetric part)<br>Anomalous Hall effect	$\rho^a = 0$	$\rho^a = 0$
Hall effect tensor R^s_{ijk}<br>(symmetric part) (spin contribution)<br>Linear magnetoresistance	$R^s_{ij1} = R^s_{ij2} = 0$,<br>$R^s_{ij3}$ no restriction	R^s_{ij1}, R^s_{ij2} no restriction,<br>$R^s_{ij3} = 0$
Hall effect tensor R^s_{ijk}<br>(symmetric part) (orbital contribution)<br>Linear magnetoresistance	$R^s = 0$	$R^s = 0$
Hall effect tensor R^a_{ijk}<br>(antisymmetric part) (spin contribution)<br>Ordinary Hall effect	$R^a = 0$	$R^a_{ij1} = R^a_{ij2} = 0$,<br>$R^a_{ij3} = 0$ no restriction
Hall effect tensor R^a_{ijk}<br>(antisymmetric part) (orbital contribution)<br>Ordinary Hall effect	no restriction	no restriction
Spin Hall resistivity tensor $\rho^{s\ k}_{ij}$<br>(symmetric part)	$\rho^{s1} = \rho^{s2} = 0$<br>$\rho^{s3}$ no restriction	ρ^{s1}, ρ^{s2} no restriction,<br>$\rho^{s3} = 0$
Spin Hall resistivity tensor $\rho^{a\ k}_{ij}$<br>(antisymmetric part)	$\rho^a = 0$	$\rho^{a1} = \rho^{a2} = 0$,<br>$\rho^{a3}$ no restriction
Ordinary Seebeck effect<br>Ordinary Peltier effect	no restriction	no restriction
Spontaneous Nernst effect<br>Spontaneous Ettingshausen effect	$\frac{1}{2}(\beta_{ij} - \pi_{ji}) = 0$<br>$\frac{1}{2}(\beta_{ji} - \pi_{ij}) = 0$	$\frac{1}{2}(\beta_{ij} - \pi_{ji}) = 0$<br>$\frac{1}{2}(\beta_{ji} - \pi_{ij}) = 0$

S1.5 Tensors of selected optical properties

Table S5: Selected examples of optical properties tensors with their Jahn symbols in the context of MPGs and SpPGs, and their transformation laws under an operation $\{U \parallel R\}$.

**Tensor description**	**Defining Equation**	**Jahn Symbol (MPG/SpPG)**	**Transformation laws (SpPG)**
Optical dielectric tensor ε^s_{ij}<br>(symmetric part)<br>Index ellipsoid	$D_i = \varepsilon_{ij} E_j$<br>$\varepsilon^s_{ij} = \frac{1}{2}(\varepsilon_{ij} + \varepsilon_{ji})$	$[\mathrm{V}^2]$	$RR\varepsilon^s$
Optical dielectric tensor ε^a_{ij}<br>(antisymmetric part)<br>Spontaneous Faraday effect	$D_i = \varepsilon_{ij} E_j$<br>$\varepsilon^a_{ij} = \frac{1}{2}(\varepsilon_{ij} - \varepsilon_{ji})$	$a\{\mathrm{V}^2\}$	$\det(U)RR\varepsilon^a$
Optical activity tensor $\gamma^s_{ij\ell}$<br>(symmetric part)<br>Spontaneous gyrotropic birefringence	$\varepsilon_{ij}(\mathbf{k}) = \varepsilon_{ij}(0) + i\gamma_{ij\ell} k_\ell$<br>$\gamma^s_{ij\ell} = \frac{1}{2}(\gamma_{ij\ell} + \gamma_{ji\ell})$	$a[\mathrm{V}^2]\mathrm{V}$	$\det(U)RRR\gamma^s$
Optical activity tensor $\gamma^a_{ij\ell}$<br>(antisymmetric part)<br>Natural optical activity	$\varepsilon_{ij}(\mathbf{k}) = \varepsilon_{ij}(0) + i\gamma_{ij\ell} k_\ell$<br>$\gamma^a_{ij\ell} = \frac{1}{2}(\gamma_{ij\ell} - \gamma_{ji\ell})$	$\{\mathrm{V}^2\}\mathrm{V}$	$RRR\gamma^a$
Pockels effect tensor r^s_{ijk}<br>(symmetric part)<br>Ordinary Pockels effect	$\varepsilon_{ij}(\mathbf{E}) = \varepsilon_{ij}(0) + r_{ijk} E_k$<br>$r^s_{ijk} = \frac{1}{2}(r_{ijk} + r_{jik})$	$[\mathrm{V}^2]\mathrm{V}$	$RRRr^s$
Pockels effect tensor r^a_{ijk}<br>(antisymmetric part)	$\varepsilon_{ij}(\mathbf{E}) = \varepsilon_{ij}(0) + r_{ijk} E_k$<br>$r^a_{ijk} = \frac{1}{2}(r_{ijk} - r_{jik})$	$a\{\mathrm{V}^2\}\mathrm{V}$	$det(U)RRRr^a$
Faraday effect tensor z^s_{ijk}<br>(symmetric part)<br>Magnetooptic Kerr effect (MOKE)	$\varepsilon_{ij}(\mathbf{H}) = \varepsilon_{ij}(0) + iz_{ijk} H_k$<br>$z^s_{ijk} = \frac{1}{2}(z_{ijk} + z_{jik})$	$ae[\mathrm{V}^2]\mathrm{V}/[\mathrm{V}^2]\mathrm{M}$	$RRUz^s$ (Spin)<br>$\det(U)\det(R)RRRz^s$<br>(Orbital)
Faraday effect tensor z^a_{ijk}<br>(antisymmetric part)<br>Ordinary Faraday effect	$\varepsilon_{ij}(\mathbf{H}) = \varepsilon_{ij}(0) + iz_{ijk} H_k$<br>$z^a_{ijk} = \frac{1}{2}(z_{ijk} - z_{jik})$	$e\{\mathrm{V}^2\}\mathrm{V}/a\{\mathrm{V}^2\}\mathrm{M}$	$\det(U)RRUz^a$ (Spin)<br>$\det(R)RRRz^a$<br>(Orbital)

S1.6 Constraints imposed by collinearity and coplanarity on optical properties

Table S6: Constraints imposed by collinearity and coplanarity on some tensors for optical properties.

**Tensor**	**Collinear structure**	**Coplanar structure**
Optical dielectric tensor ε^s_{ij} (symmetric part) Index ellipsoid	no restriction	no restriction
Optical dielectric tensor ε^a_{ij} (antisymmetric part) Spontaneous Faraday effect	$\varepsilon^a = 0$	$\varepsilon^a = 0$
Optical activity tensor γ^s_{ijk} (symmetric part) Spontaneous gyrotropic birefringence	$\gamma^s = 0$	$\gamma^s = 0$
Optical activity tensor γ^a_{ijk} (antisymmetric part) Natural optical activity	no restriction	no restriction
Pockels effect tensor r^s_{ijk} (symmetric part) Ordinary Pockels effect	no restriction	no restriction
Pockels effect tensor r^a_{ijk} (antisymmetric part)	$r^a = 0$	$r^a = 0$
Faraday effect tensor z^s_{ijk} (symmetric part, spin contribution) Magnetooptic Kerr effect (MOKE)	$z^s_{ij1} = z^s_{ij2} = 0$, z^s_{ij3} no restriction	z^s_{ij1}, z^s_{ij2} no restriction, $z^s_{ij3} = 0$
Faraday effect tensor z^s_{ijk} (symmetric part, orbital contribution) Magnetooptic Kerr effect (MOKE)	$z^s = 0$	$z^s = 0$
Faraday effect tensor z^a_{ijk} (antisymmetric part, spin contribution) Ordinary Faraday effect	$z^a = 0$	$z^a_{ij1} = z^a_{ij2} = 0$, z^a_{ij3} no restriction
Faraday effect tensor z^a_{ijk} (antisymmetric part, orbital contribution) Ordinary Faraday effect	no restriction	no restriction

S2 Nonlinear optical properties

Although there is a wide variety of nonlinear optical (NLO) properties, here we will study exclusively second-order electric-dipole processes which, when allowed, usually give the strongest signals. We will use the notation $\chi(\omega_3;\omega_2,\omega_1)$ to designate the NLO susceptibility in which input electric waves of frequencies ω_1 and ω_2 combine to produce an electric polarization at $\omega_3=\omega_2+\omega_1$, i.e.,

$$P_i(\omega_3)=\chi_{ijk}(\omega_3;\omega_2,\omega_1)E_j(\omega_2)E_k(\omega_1). \tag{1}$$

This polarization, in its turn, produces an electric field with the same frequency ω_3. Frequencies on the right of the semicolon (input waves) can be positive or negative; an input wave with negative frequency is equivalent to an output wave with a positive frequency. The frequency on the left side of the semicolon is the frequency of the output wave. It is always positive or zero.

As in the preceding cases, in order to obtain the restrictions produced by a SpPG we need the corresponding Onsager relations to find out the way these tensors behave under time reversal. In general, it turns out that the time reversal operation only gives a relation between elements of different NLO properties and is therefore not useful for tensor reduction (Gallego *et al.*, 2019). Only in one special case Onsager relations can be exploited. This is the case ($\omega_2=-\omega_1=\omega,\omega_3=0$), which corresponds to the so-called optical rectification phenomenon for which it can be shown (Gallego *et al.*, 2019) that

$$\{-1||1\}\chi_{ijk}(0;\omega,-\omega)=\chi_{ikj}(0;\omega,-\omega). \tag{2}$$

From this expression we deduce that the symmetric part of this susceptibility in the last two indices $\left[\frac{1}{2}(\chi_{ijk}(0;\omega,-\omega)+\chi_{ikj}(0;\omega,-\omega))\right]$ is even with respect to $\{-1||1\}$, and the antisymmetric part $\left[\frac{1}{2}(\chi_{ijk}(0;\omega,-\omega)-\chi_{ikj}(0;\omega,-\omega))\right]$ is odd. This behavior together with the fact that the optical rectification tensor is polar implies that the symmetric part must be of type $\mathrm{V}[\mathrm{V}^2]$ and the antisymmetric part of type $a\mathrm{V}\{\mathrm{V}^2\}$.

As has been pointed out above, there are no more tensor symmetry reductions for the general case. However, further reductions can be attained in non-dissipative media because in those materials the NLO susceptibilities possess additional symmetries. More specifically, it can be shown that the absence of dissipation implies (Pershan, 1963; Popov *et al.*, 1995; Klyshko, 2011)

$$\{-1||1\}\chi_{ijk}(\omega_3;\omega_2,\omega_1)=\left[\chi_{ijk}(\omega_3;\omega_2,\omega_1)\right]^*, \tag{3}$$

i.e., we retrieve a relation between elements of the same tensor property. Equation (3) implies that the real part behaves like V^3 and the imaginary part is of type $a\mathrm{V}^3$. The real part is interpreted physically as the contribution of the crystal lattice to χ, while the imaginary part is understood as originating from the spin arrangement (Gallego *et al.*, 2019). If in equation (3) we take the special

case of second harmonic generation, ($\omega_1 = \omega_2 = \omega, \omega_3 = 2\omega$), the tensor is symmetric in its last two indices and we arrive at the symbols V[V^2] and aV[V^2] for the real and imaginary parts, respectively. If, additionally, the medium has no dispersion, χ_{ijk} has the so-called Kleinman symmetry (Kleinman, 1962), which allows any permutation of the indices ijk in the real part (transforming then the symbol V^3 into [V^3]) and cancels out the imaginary part. Table S7 summarizes the situation in the different cases.

In this context, it is interesting to comment on a tensor with the same transformation properties as the SHG tensor in nondissipative media. This is the quadratic electrical conductivity tensor σ_{ijk}, which has recently been analyzed by Zhu *et al.* (2024). Two main contributions can be distinguished in σ_{ijk}, the so-called Berry Curvature Dipole (BCD) contribution, which is V[V2], and the Quantum Metric Dipole (QMD) contribution, which is aV[V^2]. An additional part called the Inverse Mass Dipole (IMD) contribution also appears in the odd part for the time reversal, but it is of the type a[V^3], being then a special case of the QMD contribution. It has been shown (Tsirkin & Souza, 2022) that each contribution contains an Ohmic and a Hall-type (dissipationless) part, which can be separated according to the following prescription: The Ohmic parts correspond to the fully symmetric tensors [V^3] and a[V^3], while the Hall parts are those remaining after subtracting the Ohmic parts. The separation is carried out by imposing the following 10 constraints, $\sigma_{ijk} + \sigma_{jki} + \sigma_{kij} = 0$, on the tensors since, as can be easily checked, these conditions cancel out any tensor of type [V^3] or a[V^3].

S3 Constraints on tensors for nonlinear optical susceptibilities of collinear and coplanar magnetic structures

Collinearity and coplanarity also impose restrictions on the NLO susceptibility tensors. These restrictions are easily obtained because the Jahn symbols of all the properties for SpPG are not spin dependent and can be derived from the MPG$_{\text{eff}}$ (see Table S7). Especially remarkable are the vanishing of the antisymmetric part of the optical rectification tensor, and the imaginary parts of the second-order susceptibility tensor for non-dissipative media, both for collinear and coplanar structures. Table S8 summarizes these restrictions on some tensors for second-order NLO susceptibilities

S4 Study of further properties in non-coplanar $DyVO_3$ (entry 0.106 in MAGNDATA)

We finish the study of this material with a summary of results for two rank-3 tensors: the antisymmetric $R^a_{ijk}(=-R^a_{jik})$ and symmetric $R^s_{ijk}(=R^s_{jik})$ parts of the Hall tensor. The former is responsible for the ordinary Hall effect and the latter for the linear magnetoresistance. Analogous tensors (Table 6 in the main text and Table S5) also account for the ordinary Faraday effect and

Table S7: Summary of second-order electric-dipole susceptibilities with their Jahn symbols and transformation laws in the context of SpPGs.

**Tensor description**	**Range of validity**	**Jahn Symbol (MPG and SpPG)**	**Transformation law (SpPG)**
Optical rectification $\chi_{ijk}(0;\omega,-\omega)$ (symmetric part)	General	V[V^2]	$RRR\chi^s$
Optical rectification $\chi_{ijk}(0;\omega,-\omega)$ (antisymmetric part)	General	aV{V^2}	$\det(U)RRR\chi^a$
General 2nd order susceptibility $\chi_{ijk}(\omega_3;\omega_2,\omega_1)$ (real part)	Non dissipative media	V^3	$RRR\chi^{real}$
General 2nd order susceptibility $\chi_{ijk}(\omega_3;\omega_2,\omega_1)$ (imaginary part)	Non dissipative media	aV^3	$\det(U)RRR\chi^{imag}$
Second-harmonic generation $\chi_{ijk}(2\omega;\omega,\omega)$ (real part)	Non dissipative media	V[V^2]	$RRR\chi^{real}$
Second-harmonic generation $\chi_{ijk}(2\omega;\omega,\omega)$ (imaginary part)	Non dissipative media	aV[V^2]	$\det(U)RRR\chi^{imag}$
General 2nd order susceptibility $\chi_{ijk}(\omega_3;\omega_2,\omega_1)$ (real part)	Non dissipative and dispersionless media	[V^3]	$RRR\chi^{real}$
General 2nd order susceptibility $\chi_{ijk}(\omega_3;\omega_2,\omega_1)$ (imaginary part)	Non dissipative and dispersionless media	Forbidden	Forbidden

Table S8: Constraints imposed by collinearity and coplanarity on some tensors for second-order nonlinear optical properties.

**Tensor**	**Collinear structure**	**Coplanar structure**
Optical rectification $\chi_{ijk}(0;\omega,-\omega)$ (symmetric part)	χ^s no restriction	χ^s no restriction
Optical rectification $\chi_{ijk}(0;\omega,-\omega)$ (antisymmetric part)	$\chi^a = 0$	$\chi^a = 0$
General 2nd order susceptibility $\chi_{ijk}(\omega_3;\omega_2,\omega_1)$ (real part), (Non-dissipative media)	Reχ no restriction	Reχ no restriction
General 2nd order susceptibility $\chi_{ijk}(\omega_3;\omega_2,\omega_1)$ (imaginary part), (Non-dissipative media)	Im$\chi = 0$	Im$\chi = 0$
Second-harmonic generation $\chi_{ijk}(2\omega;\omega,\omega)$ (real part), (Non-dissipative media)	Reχ no restriction	Reχ no restriction
Second-harmonic generation $\chi_{ijk}(2\omega;\omega,\omega)$ (imaginary part), (Non-dissipative media)	Im$\chi = 0$	Im$\chi = 0$
General 2nd order susceptibility $\chi_{ijk}(\omega_3;\omega_2,\omega_1)$ (real part) (Non-dissipative and dispersionless media)	Reχ no restriction	Reχ no restriction
General 2nd order susceptibility $\chi_{ijk}(\omega_3;\omega_2,\omega_1)$ (imaginary part) (Non-dissipative and dispersionless media)	Im$\chi = 0$	Im$\chi = 0$

the magneto-optic Kerr effect respectively.

For the antisymmetric part (which is even with respect to time reversal) the reduction is as follows. There are 5 independent coefficients for the MPG (R^a_{123}, R^a_{131}, R^a_{132}, R^a_{232}, R^a_{231}). On the other hand, under the SpPG, there are 3 independent coefficients for the spin contribution (R^a_{123}, R^a_{131}, R^a_{232}), and 3 independent coefficients for the orbital part (R^a_{123}, R^a_{231}, R^a_{132}). Taking both contributions together we find no further reduction under the SpPG. Regarding the symmetric part R^s_{ijk}, which is odd with respect to the time reversal, the MPG allows 10 independent coefficients (R^s_{111}, R^s_{121}, R^s_{221}, R^s_{331}, R^s_{112}, R^s_{122}, R^s_{222}, R^s_{332}, R^s_{133}, R^s_{233}). Under the SpPG only 5 of them survive for the spin contribution (R^s_{121}, R^s_{112}, R^s_{222}, R^s_{332}, R^s_{133}), and 5 for the orbital component (R^s_{111}, R^s_{122}, R^s_{133}, R^s_{221}, R^s_{331}). Taking both contributions together we only obtain one additional restriction ($R^s_{233} = 0$) under the SpPG symmetry.

In this example, it can be seen that some tensors of non-coplanar materials may not present many more constraints in the SpPGs than in the MPGs, especially if P_{SO} is the trivial group.

S5 Non-coplanar $CaFe_3Ti_4O_{12}$ (entry 3.24 in MAGNDATA)

The paramagnetic phase of $CaFe_3Ti_4O_{12}$ has space group $Im\bar{3}$ (No. 204) and the MSG of its magnetic phase is R$\bar{3}$ (OG N. 148.1.1247). The reported magnetic structure (Patino *et al.*, 2021) is shown in Fig. S1. Being a non-coplanar structure, the SpSG does not include any spin-only

subgroup (except the identity), and coincides with its nontrivial subgroup, which is labelled with the numerical index 2.148.4.1 (Chen *et al.*, 2024). Since $i_k = 4$, the SpSG cannot be minimal, despite the coincidence of the second number of the SpSG label and first number of the OG label of the MSG. In this case both G_0 and F are space groups of the same type ($R\overline{3}$), but the translation lattice of G_0 is denser than that of F. The corresponding SpPG is generated by the operations:

$$\{3_{111}||\overline{3}_{111}\}, \{2_y||1\}, \{2_z||1\},$$

while the MPG of the structure is $\bar{3}.1$, which has as single generator $\{3_{111}||\overline{3}_{111}\}$. It can be seen that the group of space operations is $\bar{3}$ in both cases, but the SpPG also includes a spin-only subgroup, $\mathrm{P_{SO}}$, generated by $\{2_y||1\}$ and $\{2_z||1\}$. This spin-only group is originated by the spin-translation group present in the SpSG, and it can be denoted as ${}^{222}1$. Then the SpPG can be written as the direct product $\mathrm{P_S} = \mathrm{P_M} \times {}^{222}1$, with $\mathrm{P_M}$ being the MSG of the structure. We examine now the form of the magnetization, the anomalous Hall effect, and the spin Hall resistivity tensor allowed by the MPG and the SpPG.

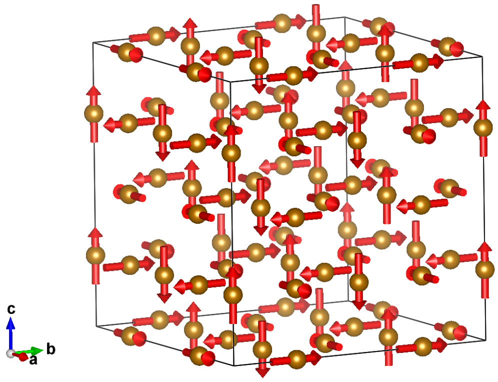

Figure S1: Magnetic structure of $CaFe_3Ti_4O_{12}$ showing only the magnetic Fe and their spin orientation.

Magnetization is allowed under the MPG, with $\mathbf{M} = (M, M, M)$, i.e., along the trigonal axis. Under the SpPG, the spin contribution to the magnetization is forbidden due to the spin-only point group, but the $\mathrm{MPG_{eff}}$ that dictates the constraints on the orbital contribution coincides with the MPG. Therefore, in the SOC-free approximation, any magnetization in the [111] direction can only be of orbital origin.

On the other hand, the anomalous Hall effect (or the spontaneous Faraday effect) is permitted both

by the MSG and SpPG, with the same form for the antisymmetric resistivity tensor:

$$\rho^a = \begin{pmatrix} 0 & \rho_{12} & -\rho_{12} \\ -\rho_{12} & 0 & \rho_{12} \\ \rho_{12} & -\rho_{12} & 0 \end{pmatrix} \quad (4)$$

or the antisymmetric optical permittivity. Thus, in this material the anomalous Hall (and the Faraday) effect may be then of geometric nature.

Finally, the MPG allows the existence of the spin Hall resistivity tensor, with 6 independent coefficients in its symmetric part:

$$\rho^{s1} = \begin{pmatrix} {\rho_{11}}^1 & {\rho_{12}}^1 & {\rho_{13}}^1 \\ {\rho_{12}}^1 & -{\rho_{11}}^1 & {\rho_{23}}^1 \\ {\rho_{13}}^1 & {\rho_{23}}^1 & 0 \end{pmatrix}, \rho^{s2} = \begin{pmatrix} {\rho_{12}}^1 & -{\rho_{11}}^1 & -{\rho_{23}}^1 \\ -{\rho_{11}}^1 & -{\rho_{12}}^1 & {\rho_{13}}^1 \\ -{\rho_{23}}^1 & {\rho_{13}}^1 & 0 \end{pmatrix}, \rho^{s3} = \begin{pmatrix} {\rho_{11}}^3 & 0 & 0 \\ 0 & {\rho_{11}}^3 & 0 \\ 0 & 0 & {\rho_{33}}^3 \end{pmatrix} \quad (5)$$

and 3 coefficients in the antisymmetric part:

$$\rho^{a1} = \begin{pmatrix} 0 & 0 & {\rho_{13}}^1 \\ 0 & 0 & {\rho_{23}}^1 \\ -{\rho_{13}}^1 & -{\rho_{23}}^1 & 0 \end{pmatrix}, \rho^{a2} = \begin{pmatrix} 0 & 0 & -{\rho_{23}}^1 \\ 0 & 0 & 0 \\ {\rho_{23}}^1 & 0 & 0 \end{pmatrix}, \rho^{a3} = \begin{pmatrix} 0 & {\rho_{12}}^3 & 0 \\ -{\rho_{12}}^3 & 0 & 0 \\ 0 & 0 & 0 \end{pmatrix} \quad (6)$$

However, according to the SpPG, all coefficients in equations (5) and (6) must be cancelled due to the constraints imposed by the spin-only group. Therefore, the whole property can only be an effect derived from the SOC. This major restriction deduced in the SpPG framework is due to the high symmetry of P_{SO}, and greatly contrasts with the preceding example.

S6 Collinear UCr_2Si_2C (entry 0.499 in MAGNDATA)

UCr_2Si_2C has a tetragonal structure with space group $P4/mmm$ (No. 123) and it is magnetically ordered at room temperature. The reported collinear magnetic structure is shown in Fig. S2. In the figure, the spin direction is taken along the x axis of the tetragonal unit cell, but in fact it is only known that the spin direction is on the basal xy plane, with its direction on the plane being experimentally undetermined (Lemoine *et al.*, 2018). For the particular spin orientation assumed in Fig. S2, and keeping the tetragonal crystallographic axes as the reference frame, the MSG of the structure can be written as $Pmm'm'$ (OG N. 47.4.350), and the corresponding MPG is $m_x m'_y m'_z$. On the other hand, the spin group symmetry, which is independent of the spin direction, is described by a collinear SpSG with nontrivial subgroup 47.123.1.1 (Chen *et al.*, 2024). The corresponding SpPG is ${}^{-1}4/{}^{1}m\,{}^{1}m\,{}^{-1}m\,{}^{\infty m}1$. The SpPG and MPG are generated by the following elements (not a minimal set in the case of the SpPG to make more explicit the relation with the MPG generators):

$$\begin{aligned} \text{SpPG} &: \{1||m_z\}, \{1||m_y\}, \{1||\bar{1}\}, \{-1||4_z\}, \{\infty_x||1\}, \{m_z||1\} \\ \text{MPG} &: \{m_z||m_z\}, \{m_y||m_y\}, \{1||\bar{1}\} \end{aligned}$$

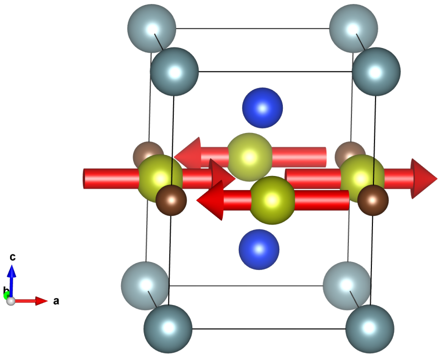

Figure S2: Magnetic structure of UCr_2Si_2C showing the spins of the Cr atoms (yellow spheres). The U, Si and C atoms are represented by gray, brown and blue colors respectively.

Hence, while the SpPG is tetragonal with respect to the lattice operations, the MPG is only orthorhombic. For other spin orientations, say x', the SpPG remains the same, with operations $\{\infty_{x'}||1\}$ instead of $\{\infty_x||1\}$, and $\{m_{z'}||1\}$ instead of $\{m_z||1\}$ (z' is perpendicular to x'), whereas the MPG is reduced to $2'_z/m'_z$. An exception occurs if x' is along [110], in which case the MPG is $m_{1\bar{1}0}m'_{110}m'_{001}$.

For the orbital contributions, the MPG_{eff} is a gray group, $4/mmm.1'$, also independent of the spin direction.

Tables S9 and S10 show various tensor properties for 3 spin orientations within the easy plane ([100], [110], and [210]), and compare them with the tensors deduced under the MPG symmetry. When there are separate tensors with spin and orbital contributions both have been added. In general, one always finds higher reduction in the SpPG, in particular for the spin along [210]. An exception is the magnetic susceptibility, which shows both spin and orbital contributions. Especially remarkable are the cancellation of the spontaneous magnetization, the antisymmetric part of the spin Hall resistivity, and the symmetric part of the spin Hall resistivity ρ^{s3} under the SpPG symmetry for all spin orientations. Note that as the SpPG is mathematically the same regardless of the spin direction, the number of resulting independent coefficients in each tensor is the same for all three spin orientations (Table S10), which is not the case when the MPG is considered.

Table S9: Examples of symmetry-adapted tensors under MPG and SpPG symmetries for three different spin orientations in the collinear material UCr_2Si_2C.

**Spin direction**	**Tensor property**	**MPG**	**SpPG**
100	Magnetization	$(M_1, 0, 0)$	$(0, 0, 0)$
110	Magnetization	$(M_1, -M_1, 0)$	$(0, 0, 0)$
210	Magnetization	$(M_1, M_2, 0)$	$(0, 0, 0)$
100	Magnetic susceptibility	$\begin{pmatrix} \chi_{11} & 0 & 0 \\ 0 & \chi_{22} & 0 \\ 0 & 0 & \chi_{33} \end{pmatrix}$	$\begin{pmatrix} \chi_{11} & 0 & 0 \\ 0 & \chi_{22} & 0 \\ 0 & 0 & \chi_{33} \end{pmatrix}$
110	Magnetic susceptibility	$\begin{pmatrix} \chi_{11} & \chi_{12} & 0 \\ \chi_{12} & \chi_{11} & 0 \\ 0 & 0 & \chi_{33} \end{pmatrix}$	$\begin{pmatrix} \chi_{11} & \chi_{12} & 0 \\ \chi_{12} & \chi_{11} & 0 \\ 0 & 0 & \chi_{33} \end{pmatrix}$
210	Magnetic susceptibility	$\begin{pmatrix} \chi_{11} & \chi_{12} & 0 \\ \chi_{12} & \chi_{22} & 0 \\ 0 & 0 & \chi_{33} \end{pmatrix}$	$\begin{pmatrix} \chi_{11} & \chi_{12} & 0 \\ \chi_{12} & \chi_{22} & 0 \\ 0 & 0 & \chi_{33} \end{pmatrix}$
100	Electric resistivity	$\begin{pmatrix} \rho_{11} & 0 & 0 \\ 0 & \rho_{22} & \rho_{23} \\ 0 & -\rho_{23} & \rho_{33} \end{pmatrix}$	$\begin{pmatrix} \rho_{11} & 0 & 0 \\ 0 & \rho_{11} & 0 \\ 0 & 0 & \rho_{33} \end{pmatrix}$
110<br>210	Electric resistivity	$\begin{pmatrix} \rho_{11} & \rho_{12} & \rho_{13} \\ \rho_{12} & \rho_{11} & \rho_{23} \\ -\rho_{13} & -\rho_{23} & \rho_{33} \end{pmatrix}$	$\begin{pmatrix} \rho_{11} & 0 & 0 \\ 0 & \rho_{11} & 0 \\ 0 & 0 & \rho_{33} \end{pmatrix}$

Table S10: Symmetry-adapted tensors under MPG and SpPG symmetries of the symmetric and antisymmetric parts of the spin Hall resistivity (SHR) tensor ${\rho_{ij}}^{k}$ for 3 different spin orientations in the collinear material UCr_2Si_2C.

**SHR tensor**	**MPG**	**SpPG**
Symmetric part [100]	$\begin{pmatrix} {\rho_{11}}^{1} & 0 & 0 \\ 0 & {\rho_{22}}^{1} & 0 \\ 0 & 0 & {\rho_{33}}^{1} \end{pmatrix}, \begin{pmatrix} 0 & {\rho_{12}}^{2} & 0 \\ {\rho_{12}}^{2} & 0 & 0 \\ 0 & 0 & 0 \end{pmatrix}, \begin{pmatrix} 0 & 0 & {\rho_{13}}^{3} \\ 0 & 0 & 0 \\ {\rho_{13}}^{3} & 0 & 0 \end{pmatrix}$	$\begin{pmatrix} {\rho_{11}}^{1} & 0 & 0 \\ 0 & -{\rho_{11}}^{1} & 0 \\ 0 & 0 & 0 \end{pmatrix}, \rho^2 = \rho^3 = 0$
Antiymmetric part [100]	$\begin{pmatrix} 0 & 0 & 0 \\ 0 & 0 & {\rho_{23}}^{1} \\ 0 & -{\rho_{23}}^{1} & 0 \end{pmatrix}, \begin{pmatrix} 0 & 0 & {\rho_{13}}^{2} \\ 0 & 0 & 0 \\ -{\rho_{13}}^{2} & 0 & 0 \end{pmatrix}, \begin{pmatrix} 0 & {\rho_{12}}^{3} & 0 \\ -{\rho_{12}}^{3} & 0 & 0 \\ 0 & 0 & 0 \end{pmatrix}$	$\rho^1 = \rho^2 = \rho^3 = 0$
Symmetric part [110]	$\begin{pmatrix} {\rho_{11}}^{1} & {\rho_{12}}^{1} & 0 \\ {\rho_{12}}^{1} & {\rho_{22}}^{1} & 0 \\ 0 & 0 & {\rho_{33}}^{1} \end{pmatrix}, \begin{pmatrix} -{\rho_{22}}^{1} & -{\rho_{12}}^{1} & 0 \\ -{\rho_{12}}^{1} & -{\rho_{11}}^{1} & 0 \\ 0 & 0 & -{\rho_{33}}^{1} \end{pmatrix}, \begin{pmatrix} 0 & 0 & {\rho_{13}}^{3} \\ 0 & 0 & -{\rho_{13}}^{3} \\ {\rho_{13}}^{3} & -{\rho_{13}}^{3} & 0 \end{pmatrix}$	$\begin{pmatrix} {\rho_{11}}^{1} & 0 & 0 \\ 0 & -{\rho_{11}}^{1} & 0 \\ 0 & 0 & 0 \end{pmatrix}, \begin{pmatrix} {\rho_{11}}^{1} & 0 & 0 \\ 0 & -{\rho_{11}}^{1} & 0 \\ 0 & 0 & 0 \end{pmatrix}, \rho^3 = 0$
Antiymmetric part [110]	$\begin{pmatrix} 0 & 0 & {\rho_{13}}^{1} \\ 0 & 0 & {\rho_{23}}^{1} \\ -{\rho_{13}}^{1} & -{\rho_{23}}^{1} & 0 \end{pmatrix}, \begin{pmatrix} 0 & 0 & -{\rho_{23}}^{1} \\ 0 & 0 & -{\rho_{13}}^{1} \\ {\rho_{23}}^{1} & {\rho_{13}}^{1} & 0 \end{pmatrix}, \begin{pmatrix} 0 & {\rho_{12}}^{3} & 0 \\ -{\rho_{12}}^{3} & 0 & 0 \\ 0 & 0 & 0 \end{pmatrix}$	$\rho^1 = \rho^2 = \rho^3 = 0$
Symmetric part [210]	$\begin{pmatrix} {\rho_{11}}^{1} & {\rho_{12}}^{1} & 0 \\ {\rho_{12}}^{1} & {\rho_{22}}^{1} & 0 \\ 0 & 0 & {\rho_{33}}^{1} \end{pmatrix}, \begin{pmatrix} {\rho_{11}}^{2} & {\rho_{12}}^{2} & 0 \\ {\rho_{12}}^{2} & {\rho_{22}}^{2} & 0 \\ 0 & 0 & {\rho_{33}}^{2} \end{pmatrix}, \begin{pmatrix} 0 & 0 & {\rho_{13}}^{3} \\ 0 & 0 & {\rho_{23}}^{3} \\ {\rho_{13}}^{3} & {\rho_{23}}^{3} & 0 \end{pmatrix}$	$\begin{pmatrix} {\rho_{11}}^{1} & 0 & 0 \\ 0 & -{\rho_{11}}^{1} & 0 \\ 0 & 0 & 0 \end{pmatrix}, \begin{pmatrix} \frac{{\rho_{11}}^{1}}{2} & 0 & 0 \\ 0 & -\frac{{\rho_{11}}^{1}}{2} & 0 \\ 0 & 0 & 0 \end{pmatrix}, \rho^3 = 0$
Antiymmetric part [210]	$\begin{pmatrix} 0 & 0 & {\rho_{13}}^{1} \\ 0 & 0 & {\rho_{23}}^{1} \\ -{\rho_{13}}^{1} & -{\rho_{23}}^{1} & 0 \end{pmatrix}, \begin{pmatrix} 0 & 0 & {\rho_{13}}^{2} \\ 0 & 0 & {\rho_{23}}^{2} \\ -{\rho_{13}}^{2} & -{\rho_{23}}^{2} & 0 \end{pmatrix}, \begin{pmatrix} 0 & {\rho_{12}}^{3} & 0 \\ -{\rho_{12}}^{3} & 0 & 0 \\ 0 & 0 & 0 \end{pmatrix}$	$\rho^1 = \rho^2 = \rho^3 = 0$

References

Chen, X., Ren, J., Zhu, Y., Yu, Y., Zhang, A., Liu, P., Li, J., Liu, Y., Li, C. & Liu, Q. (2024). *Phys. Rev. X.* **14**(3), 031038.

Gallego, S. V., Etxebarria, J., Elcoro, L., Tasci, E. S. & Perez-Mato, J. M. (2019). *Acta Crystallogr. A Found. Adv.* **75**(3), 438–447.
Kleinman, D. A. (1962). *Phys. Rev.* **126**(6), 1977–1979.
Klyshko, D. N. (2011). *Physical foundations of quantum electronics.* Singapore, Singapore: World Scientific Publishing.
Lemoine, P., Vernière, A., Pasturel, M., Venturini, G. & Malaman, B. (2018). *Inorg. Chem.* **57**(5), 2546–2557.
Patino, M. A., Romero, F. D., Goto, M., Saito, T., Orlandi, F., Manuel, P., Szabó, A., Kayser, P., Hong, K. H., Alharbi, K. N., Attfield, J. P. & Shimakawa, Y. (2021). *Phys. Rev. Res.* **3**(4), 043208.
Pershan, P. S. (1963). *Phys. Rev.* **130**(3), 919–929.
Popov, S., Svirko, Y. & Zheludev, N. (1995). *Susceptibility Tensors for Nonlinear Optics.* Series in Optics and Optoelectronics. Taylor & Francis.
Tsirkin, S. S. & Souza, I. (2022). *SciPost Phys. Core*, **5**, 039.
Zhu, H., Li, J., Chen, X., Yu, Y. & Liu, Q. (2024). Magnetic geometry to quantum geometry nonlinear transports.
https://arxiv.org/abs/2406.03738
